# Oleuropein enhances proteasomal activity and reduces mutant huntingtin-induced cytotoxicity

**DOI:** 10.3389/fphar.2024.1459909

**Published:** 2024-09-13

**Authors:** Zih-Ning Huang, Sin-Yi Lee, Jie-Mao Chen, Zih-Ting Huang, Lu-Shiun Her

**Affiliations:** Department of Life Sciences, National Cheng Kung University, Tainan, Taiwan

**Keywords:** Huntington’s disease, mutant huntingtin aggregates, oleuropein, proteasome, ROS

## Abstract

**Introduction:**

Huntington’s disease (HD) is a hereditary neurodegenerative disorder that primarily affects the striatum, a brain region responsible for movement control. The disease is characterized by the mutant huntingtin (mHtt) proteins with an extended polyQ stretch, which are prone to aggregation. These mHtt aggregates accumulate in neurons and are the primary cause of the neuropathology associated with HD. To date, no effective cure for HD has been developed.

**Methods:**

The immortalized ST*Hdh* ^*Q111/Q111*^ striatal cell line, the mHtt-transfected wild-type ST*Hdh* ^*Q7/Q7*^ striatal cell line, and N2a cells were used as Huntington's disease cell models. Flow cytometry was used to assess cellular reactive oxygen species and transfection efficiency. The CCK-8 assay was used to measure cell viability, while fluorescence microscopy was used to quantify aggregates. Immunoblotting analyses were used to evaluate the effects on protein expression.

**Results:**

Polyphenols are natural antioxidants that offer neuroprotection in neurological disorders. In this study, we provide evidence that oleuropein, the primary polyphenol in olive leaves and olive oil, enhances cell viability in HD cell models, including. ST*Hdh* ^*Q7/Q7*^ST*Hdh* ^*Q7/Q7*^ striatal cells, N2a cells ectopically expressing the truncated mHtt, and ST*Hdh* ^*Q111/Q111*^ striatal cells expressing the full-length mHtt. Oleuropein effectively reduced both soluble and aggregated forms of mHtt protein in these HD model cells. Notably, the reduction of mHtt aggregates associated with oleuropein was linked to increased proteasome activity rather than changes in autophagic flux. Oleuropein seems to modulate proteasome activity through an unidentified pathway, as it did not affect the 20S proteasome catalytic β subunits, the proteasome regulator PA28γ, or multiple MAPK pathways.

**Discussion:**

We demonstrated that oleuropein enhances the degradation of mHtt by increasing proteasomal protease activities and alleviates mHtt-induced cytotoxicity. Hence, we propose that oleuropein and potentially other polyphenols hold promise as a candidate for alleviating Huntington's disease.

## 1 Introduction

Huntington’s disease (HD), an autosomal dominant neurodegenerative disorder, is caused by an expansion of the CAG trinucleotide repeats in the exon one of the huntingtin (Htt) gene (MacDonald et al., 1993). The expression of mutant Htt (mHtt) induces cell death in neuronal predisposing mHtt to misfolding and subsequent aggregation. Compelling evidence has linked aggregation of mHtt to striatal neuronal death, and this is recognized as one of the main pathogenic factors in HD (Davies et al., 1997; DiFiglia et al., 1997; Martín-Aparicio et al., 2001; Intihar et al., 2019; Layburn et al., 2022). The cellular dysfunctions associated with mHtt aggregates include proteasomal and mitochondrial dysfunctions and decreased cell viability (Yin et al., 2016; Soares et al., 2019; Fão and Rego, 2021). Hence, neuroprotective treatments such as anti-aggregation compounds, aggregate clearance strategies, and gene-silencing approaches to remove mHtt aggregates have been developed as potential therapeutic strategies for HD patients (Martín-Aparicio et al., 2001; Soares et al., 2019; Huang et al., 2021; Rippin et al., 2021; Zhang et al., 2021; Hommen et al., 2022; Ferlazzo et al., 2023; Jain and Roy, 2023; Yang et al., 2023; Jain et al., 2024).

Recently, microRNA (miRNA) administration has been reported to reduce mHtt aggregates and slow the disease progression of HD model mice (Cheng et al., 2013a; Ban et al., 2017; Chang et al., 2021; Spronck et al., 2021; Kotowska-Zimmer et al., 2022; Chan et al., 2023). However, delivery of the miRNA requires injection into the central nervous system, which is challenging for practical purposes and has ethical concerns. In addition, it is difficult to assess the efficacy because miRNAs are generally unstable. Therefore, whether miRNAs can be used as therapeutic agents remains debatable.

The autophagy-lysosome pathway and the Ubiquitin Proteasome System are the two major cellular proteolytic pathways (Watanabe et al., 2020; Rusilowicz-Jones et al., 2022). Inhibition of either the autophagy or proteasome impairs the degradation of mHtt protein and leads to the accumulation of mHtt aggregates (Koyuncu et al., 2017; Valionyte et al., 2020; Folger and Wang, 2021). For example, activation of the UPS activity by inhibiting the p38 MAPK pathway reduces both the soluble and aggregated forms of mHtt and increases cell viability in the ST*Hdh* striatal HD model cells (Huang et al., 2021). Also, activation of the autophagy pathway promotes clearance of mHtt aggregates and increases cell viability in human neuroblastoma SH-SY5Y cells, mouse embryonic fibroblast, and the Hdh140Q knock-in HD model mice [(Sarkar et al., 2007; Chaudhary et al., 2021; Long et al., 2022) and reviewed in Valionyte et al. (2020); Folger and Wang (2021)]. Therefore, seeking small molecules that can enhance autophagy and/or proteasome activities is a logical strategy to achieve clearance of the mHtt protein and ameliorate HD phenotype.

Oleuropein is the major phenolic compound present in olive oil and olive leaves (Omar, 2010). Oleuropein is highly antioxidative and has been demonstrated to protect against neurodegenerative diseases and cardiovascular and metabolic disorders [(Rigacci et al., 2015; Achour et al., 2016; Brunetti et al., 2020; Butt et al., 2021; Leri et al., 2021) and review in Micheli et al. (2023)]. In addition to its antioxidative properties, oleuropein increases autophagy and proteasomal activity (Katsiki et al., 2007; Huang and Chen, 2009; El Demerdash et al., 2021). Here, we explored the potential of oleuropein to improve cell viability and its effect on reducing mHtt aggregates in HD model cells. We also investigated whether oleuropein affects HD model cells’ proteasome and autophagy activities. Our data provided evidence to show that oleuropein has neuroprotective effects.

## 2 Materials and methods

### 2.1 Cell line, cell culture, and drug treatments

The immortalized wild-type ST*Hdh^Q7/Q7^* striatal cell line (RRID: CVCL_M590, Coriell ID: CH00097) and the mutant ST*Hdh* ^*Q111/Q111*^ striatal cell line (RRID: CVCL_M591, Coriell ID: CH00095) were obtained from Coriell (United States of America) (Trettel et al., 2000). Mouse neuroblastoma Neuro-2a (N2a) cell line was purchased from Bioresource Collection and Research Center, Taiwan (RRID: CVCL_0470, BCRC, Cat#60026). Cell culture of the immortalized ST*Hdh* striatal cells and N2a was carried out as described previously (Huang et al., 2017; Huang and Her, 2017; Huang et al., 2021). Oleuropein (Cat#12247, Sigma, United States of America) was dissolved in DMF to make a 50 mg/mL stock solution. The cells were incubated with oleuropein at 0.5 μg/mL, 2 μg/mL, or 5 μg/mL for 24 or 48 h, as described in Figure legends. Before treatment, the 50 mg/mL oleuropein stock was first diluted in 1:1,000 in cell medium to generate a 50 μg/mL working solution. The 50 μg/mL oleuropein working solution was further diluted in a cell medium to achieve the desired concentration. The DMF concentration in the cell medium was kept at 0.1% throughout the oleuropein experiments. To assess autophagic flux, N2a cells and ST*Hdh* striatal cells were treated with 200 nM Bafilomycin A1 (Cat#B1793, Sigma, United States of America) for 6 h to block the fusion of the autophagosome with a lysosome (Klionsky et al., 2021). To assess the stability of soluble Htt, N2a cells and ST*Hdh^Q7/Q7^* striatal cells were incubated with 100 μg/mL cycloheximide (Cat#C4859, Sigma, United States of America) for 24 h to block protein synthesis (Schneider-Poetsch et al., 2010; #298767).

### 2.2 DNA constructs and transfection

The pHttQP25-GFP and pHttQP72-GFP expression vectors, containing the exon one of human huntingtin and GFP fusion proteins with 25 or 72 CAG repeats, respectively, were obtained from the CHDI/High Q Foundation (United States of America). The pcDNA3.1-mCherry vector, containing the mCherry fluorescent protein coding sequence, was provided by Dr. Roger Y. Tsien of the University of California, San Diego. The p3xFLAG-QP25 and p3xFLAG-QP103 expression vectors were constructed by inserting the exon one of human huntingtin with 25 and 103 CAG repeats, respectively, into the p3xFLAG-CMV-10 expression vector to generate the FLAG-tagged truncated human huntingtin with 25 or 103 glutamines, respectively (Sigma). The pEBV-HttQ120F expression vector, containing the full-length human huntingtin with 120 CAG repeat coding sequence, was kindly provided by Dr. Xiao-Jiang Li of Jinan University in Guangzhou, China (Zhou et al., 2003). For transfection of the indicated plasmids, the immortalized ST*Hdh* striatal cells and N2a cells were incubated with Lipofectamine 3,000 (Cat#L3000015, Invitrogen, United States of America) according to the manufacturer’s protocol.

### 2.3 Transfection efficiency assessment

Forty-eight hours after transfection of ST*Hdh* ^*Q7/Q7*^ striatal cells with HttQP25-GFP or HttQP72-GFP plasmid, the cells were collected for GFP fluorescence analysis using Attune NxT flow cytometry (Thermo Fisher Scientific, United States of America). The cell population was gated by FSC and SSC channels, and the GFP signals were detected in the BL1 channel. The GFP positive signal was gated by the fluorescence intensity over 10^3^. The transfection efficiency was calculated by analyzing the percentage of GFP-positive cells out of the total number of cells (set as 100%).

### 2.4 CCK-8 cell viability assay

Cells were pre-incubated with DMF or oleuropein at the concentration of 0.5 μg/mL, 2 μg/mL or 5 μg/mL for 24 h before treatment with 100 μM tBHP (Cat#B2633, Sigma, United States of America) for 4 h. CCK-8 cell viability assays were carried out as described before (Huang et al., 2021).

### 2.5 Analysis of cellular reactive oxygen species (ROS) and superoxide (SO) levels

For ROS and SO detection, the immortalized ST*Hdh* striatal cells and N2a cells were seeded in a 6 cm petri dish as described before (Huang et al., 2017). The levels of ROS and SO were determined by Flow cytometry, the BD Biosciences FACS Caliber system (San Jose, CA), with Total ROS/SO detection kit (Cat#ENZ-51010, Enzo, United States of America) as suggested by the manufacturer’s instructions. Briefly, cells were washed with 1X phosphate buffered saline (PBS, pH7.4) and incubated with the ROS and SO detection solutions at 37°C for 30 min in the dark. ROS (Green) signals were detected in the FL1 channel, and signals produced by SO (Orange) were detected in the FL2 channel. Detected cells will appear in the upper left and upper right quadrants of a log FL1 (X-axis) versus a log FL2 (Y-axis) dot plot. For each sample, at least 1 × 10^4^ cells were analyzed. The percentage of ROS-positive and SO-positive cells was calculated using the FlowJo (ver.10.7.1) software. Quantification data were derived from three biological replicates.

### 2.6 Fluorescence microscopy imaging and quantification of aggregate formation

Fluorescence microscopy imaging was carried out as described previously (Huang et al., 2017; Huang et al., 2021). The mutant Htt-GFP aggregates were visualized with Eclipse Ti epifluorescence inverted microscope (Nikon, Japan). Aggregates were defined as a clear region of dense GFP signal as described previously (Juenemann et al., 2011; Cheng et al., 2013b; Huang and Her, 2017). The co-transfected red mCherry fluorescence signal was used to calculate the total number of transfected cells as a control for transfection efficiency. The percentage of cells containing the aggregates was calculated as the ratio of the number of red cells (mCherry transfected control) with mutant Htt-GFP aggregates/total number of red cells counted (set as 100%). More than 100 red mCherry fluorescence signal-positive cells were counted in each experiment. Data were average obtained from three independent experiments.

### 2.7 Antibodies and immunoblotting analysis

Antibodies used in this study are: anti-Htt (MAB2166, 1:1,000; Millipore Bioscience Research Reagents), anti-Htt (mEM48, MAB5374, 1:500; Millipore Bioscience Research Reagents), anti-p62 (ab56416, 1:1,000; Abcam), anti-LC3B (ab48394, 1:500; Abcam), anti-Proteasome 20S β1 (BML-PW8140, 1:1,000; Enzo), anti-Proteasome 20S β2 (BML-PW8145, 1:1,000; Enzo), anti-Proteasome 20S β5 (BML-PW8895, 1:1,000; Enzo), anti-PA28γ (PSME3) (GTX106722, 1:2000; GeneTex), anti-Phospho-p38 MAPK (Thr80/Tyr182) (#4511, 1:1,000, Cell Signaling), anti-p38 MAPK (#9212, 1:1,000, Cell Signaling), anti-Phospho-Nrf2 (Phospho S40) (ab76026, 1:5,000, Abcam), anti-Nrf2 (ab62352, 1:1,000, Abcam), anti-Phospho-p44/p42 MAPK (Thr202/Tyr204) (#4370, 1:1,000, Cell Signaling), anti-p44/p42 (#4696, 1:1,000, Cell Signaling), anti-Phospho-SAPK/JNK (Thr183/Tyr182) (#4668, 1:1,000, Cell Signaling), anti-SAPK/JNK (#9252, 1:1,000, Cell Signaling), anti-Phospho-ROCK2 (phosphor Ser1366) (GTX122651, 1:1,000, GeneTex), anti-ROCK2 (GTX108247, 1:1,000, GeneTex), anti-Tubulin (T6074, 1:5,000; Sigma), and anti-Actin (MAB1501, 1:1,000; Millipore Bioscience Research Reagents). Immunoblotting assays were carried out as described previously (Huang and Her, 2017; Huang et al., 2021). Proteins were detected using the enhanced chemiluminescence (ECL) assay-Western Lightning^®^ ECL Pro (Cat#NEL105001EA, PerkinElmer, United States of America). The signals were captured by iBright FL1500 Imaging System (Thermo Fisher Scientific) and UVP ChemStudio PLUS Imaging Systems (Analytik Jena), respectively. Proteins quantification and background correction were analyzed using the iBright analysis software (ver.4.0.1, Thermo Fisher Scientific) and the VisionWorks (ver.9.1.20063.7760, UVP ChemStudio PLUS Imaging Systems analysis software, Analytik Jena), respectively.

### 2.8 Proteasome core protease activity assay

The proteasome core protease activity assay was carried out as described previously (Huang et al., 2021). Cell lysates were prepared with a Tris-based buffer (50 mM Tris pH 7.4, 1 mM EDTA, 2 mM ATP, and 1% Triton). Proteasome activity assay was conducted in a 96-well plate format. For each well, 10 μg of protein lysate was incubated with the three different fluorogenic substrates separately: 50 μM of Suc-Leu-Leu-Val-Tyr-7-amino-4-methylcoumarin (Suc-LLVY-AMC, Cat# BML-P802-0,005, Enzo Life Sciences, United States of America) was used for chymotrypsin-like activity assay; 25 μM of Boc-Leu-Arg-Arg-7-amino-4-methylcoumarin (Boc-LRR-AMC, Cat# BML-BW8515-0,005, Enzo Life Sciences, United States of America) was used for trypsin-like activity assay; and 50 μM of Z-Leu-Leu-Glu-7-amino-4-methylcoumarin (Z-LLE-AMC, Cat# BML-ZW9345-0,005, Enzo Life Sciences, United States of America) as used for the caspase-like activity assay. To detect free AMC released during the enzymatic reaction, the protease-cleaved fluorogenic products were excited at 360 nm, and the resulting fluorescence emission was detected at 460 nm using a fluorescent plate reader (TECAN Infinite M200 fluorescent plate reader, Tecan Trading AG, Switzerland). The release rate of AMC was measured at 37°C by recording the emitted fluorescence signal every 5 min for a total of 120 min.

### 2.9 Statistical analysis

Determination of the statistical differences between groups was carried out using the Student’s t-test, which was reported as a *p*-value. Data showed significant differences with *p* < 0.05 are labeled with one asterisk (*); with *p* < 0.01 are labeled with two asterisks (**); with *p* < 0.005 are labeled with three asterisks (***); *N.S.*: not significant. The experimental results from three independent experiments were calculated and presented as mean ± standard error of the mean (SEM).

## 3 Results

### 3.1 Oleuropein ameliorates mutant htt-induced cytotoxicity

Knowing that oleuropein provides neuroprotection in both cell and animal models of Alzheimer’s disease (Leri et al., 2021; Marianetti et al., 2022), we were interested in asking whether oleuropein could also protect neurons in HD model cells. In this study, we used the ST*Hdh* ^*Q7/Q7*^ striatal cell as our system because the medium spiny neuron in the striatum is the most affected in HD patients, and the ST*Hdh* ^*Q7/Q7*^ striatal cells, an immortalized cell line derived from the striatal medium spiny neurons of the wild-type *Hdh* ^*Q7/Q7*^ mice, are commonly used (Trettel et al., 2000; Pal et al., 2006; Jin et al., 2013; Yin et al., 2016; Okada et al., 2021).

**FIGURE 1:**
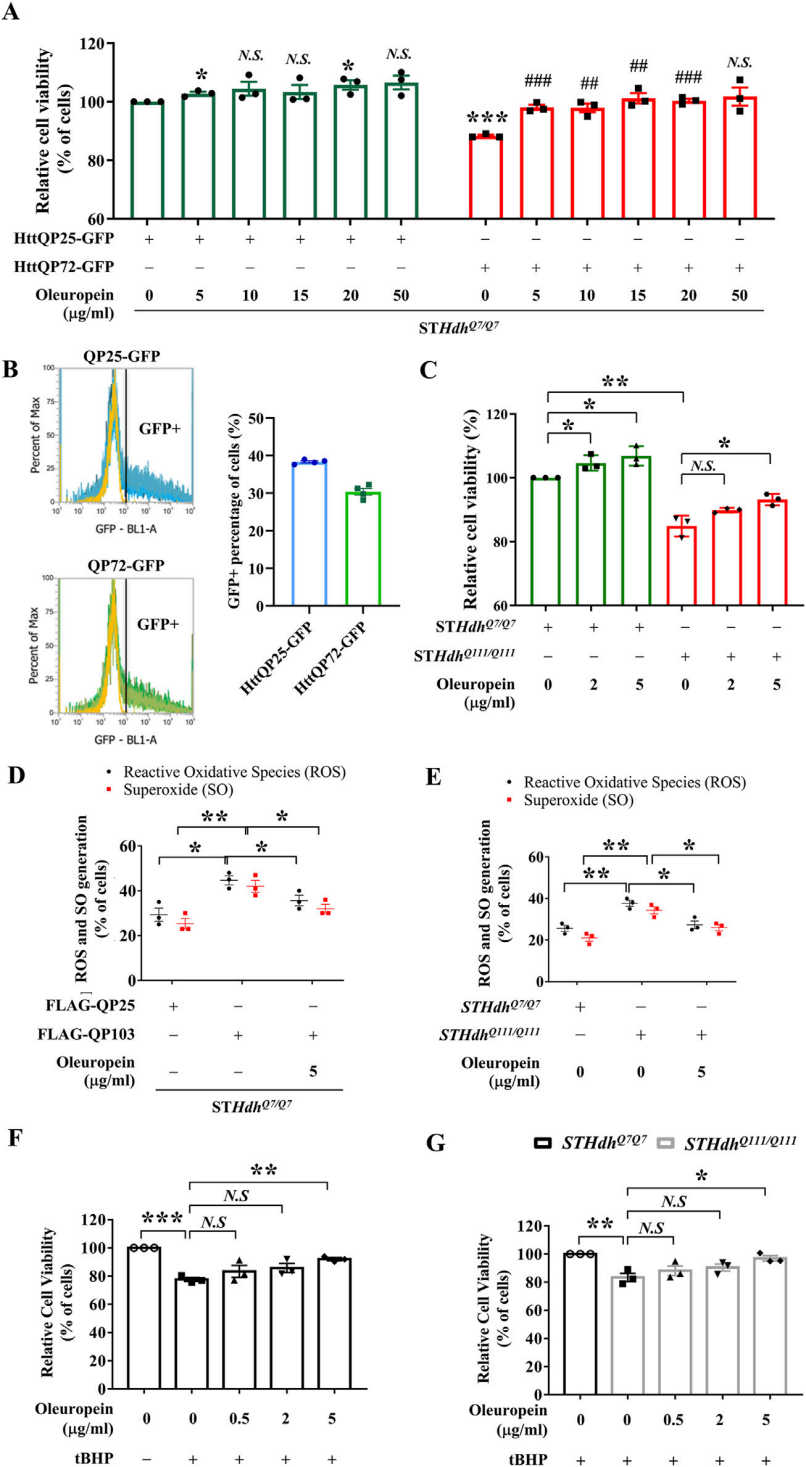
Oleuropein ameliorates mHtt-induced cytotoxicity. **(A)** Cell viability analysis of ST*Hdh* ^*Q7/Q7*^ striatal cells transfected with the HttQP25-GFP or HttQP72-GFP plasmid. 24 h after transfection, cells were incubated with oleuropein at the indicated concentration for another 24 h. Cells without treatment were used as the negative control. Data showing a significant difference compared to the HttQP25-GFP transfected cells treated with 0 μg/ml oleuropein is labeled with an asterisk (*); Data showing a significant difference compared to the HttQP72-GFP transfected cells treated with 0 μg/ml oleuropein is labeled with a pound sign (#). **(B)** Transfection efficiency analysis of the ST*Hdh* ^*Q7/Q7*^ striatal cells transfected with the HttQP25-GFP or HttQP72-GFP plasmid using flow cytometry analysis. **(C)** Cell viability analysis of ST*Hdh* ^*Q7/Q7*^ and ST*Hdh* ^*Q111/Q111*^ striatal cells after incubation with oleuropein at the indicated concentration for 24 h. Cells without treatment were used as the negative control. **(D)** Flow cytometric analysis of reactive oxygen species (ROS) and superoxide (SO) levels in ST*Hdh* ^*Q7/Q7*^ striatal cells transfected with the FLAG-QP25 or FLAG-QP103 plasmid in the presence or absence of oleuropein. The percentage of the cells with positive ROS or SO signal was quantified. Cells were incubated with oleuropein at the indicated concentration for 24 h. **(E)** Flow cytometric analysis of ROS and SO levels in ST*Hdh* ^*Q7/Q7*^ and ST*Hdh* ^*Q111/Q111*^ striatal cells plasmid in the absence or presence of oleuropein at the indicated concentration for 24 h. **(F)** Cell viability analysis of tBHP (100 μM)-treated ST*Hdh* ^*Q7/Q7*^ striatal cells without or with oleuropein pre-incubation at the indicated concentration for 24 h. **(G)** Cell viability analysis in tBHP-treated ST*Hdh* ^*Q7/Q7*^ and ST*Hdh* ^*Q111/Q111*^ striatal cells in the absence or presence of incubation with oleuropein at the indicated concentration for 24 h. Data from three independent experiments are presented as mean normalized units ± SEM. Data showing significant differences are labeled as follows: p < 0.05 with one asterisk (*), p < 0.01 with two asterisks (**), and *p* < 0.005 with three asterisks (***).

To establish an HD cell model, a truncated mutant huntingtin (mHtt)-GFP reporter protein with a 72 polyQ stretch (GFP-QP72) was expressed in ST*Hdh* ^*Q7/Q7*^ striatal cells. This truncated mHtt protein, encoded by the Htt exon 1 with expanded CAG repeats, is a cleavage product of the full-length mHtt protein and has been shown to form aggregates and induce cytotoxicity (Lunkes et al., 2002; Yang et al., 2020). Ectopic expression of the truncated wild-type Htt (encoded by the Htt exon 1)-GFP reporter (GFP-QP25) was used as a control. The transfection efficiency was assessed by flow cytometry as the percentage of cells displaying a GFP-positive signal. The transfection efficiency of HttQP25-GFP and HttQP72-GFP in ST*Hdh* ^*Q7/Q7*^ striatal cells was 38.33% and 30.37%, respectively (Figure 1B). Cell viability was measured 24 h after oleuropein incubation. As predicted, ectopic expression of the mutant GFP-QP72 mHtt protein decreased the cell viability of ST*Hdh* ^*Q7/Q7*^ striatal cells (from 100% to 88%; *p* < 0.0001, Figure 1A). Oleuropein at 5, 10, 15, 20, or 50 μg/mL was used to treat the ST*Hdh* ^*Q7/Q7*^ striatal cells expressing the mutant GFP-QP72 mHtt protein. Oleuropein treatment improved the cell viability of the ST*Hdh* ^*Q7/Q7*^ striatal cells expressing GFP-QP72 mHtt protein at all of the concentrations tested (Figure 1A). However, increasing oleuropein above a concentration of 5 μg/mL oleuropein (from 88% to 98%, *p =* 0.0005, Figure 1A) did not provide further protection (Figure 1A). Therefore, we used five ug/mL oleuropein for the rest of the experiments.

To test whether oleuropein-associated neuroprotection is a general or cell line-specific effect, we extended this study to the mouse N2a neuronal cells. Similar to the ST*Hdh* ^*Q7/Q7*^ striatal cells, treatment of five ug/mL oleuropein increased the cell viability of N2a cells expressing the mutant GFP-QP72 protein (from 86.41% to 92.41%; *p* = 0.034, Supplementary Figure S1). Oleuropein did not affect the cell viability of the N2a cells expressing the GFP-QP25 protein (from 100% to 101.6%; *p* > 0.05, Supplementary Figure S1C).

Because cellular abnormalities of the mutant ST*Hdh* ^*Q111/Q111*^ striatal cells that express the endogenous full-length mutant Htt protein resemble damaged neurons reported in HD patients (Trettel et al., 2000; Pal et al., 2006; Jin et al., 2013; Yin et al., 2016; Okada et al., 2021), we tested the effect of oleuropein on the cytotoxicity of mutant ST*Hdh* ^*Q111/Q111*^ striatal cells. Consistent with previous studies, the mutant ST*Hdh* ^*Q111/Q111*^ striatal cells had reduced cell viability as compared to the wild-type ST*Hdh* ^*Q7/Q7*^ striatal cells (from 100% to 84.91%; *p* = 0.0013, Figure 1C). Notably, oleuropein at the 5 μg/mL concentration increased the cell viability of the mutant ST*Hdh* ^*Q111/Q111*^ striatal cells (from 84.91% to 93.19%; *p* = 0.0182, Figure 1C).

Moreover, extending the duration of oleuropein treatment led to an improved protective effect. Cell viability of ST*Hdh* ^*Q7/Q7*^ striatal cells transfected with the HttQP72-GFP plasmid was increased by 25% following 48 h of 5 μg/mL oleuropein treatment, starting 48 h post-transfection (from 81.14% to 106%; *p* = 0.0009, Supplementary Figure S1A). Cell viability of mutant ST*Hdh* ^*Q111/Q111*^ striatal cells was increased by 28% following 48 h of 5 μg/mL oleuropein treatment (from 79.99% to 108.6%; *p* = 0.0001, Supplementary Figure S1B). Taken together, our data demonstrated that oleuropein reduced the cytotoxicity induced by the GFP-QP72 mHtt protein in the ST*Hdh* ^*Q7/Q7*^ striatal and N2a cells. Moreover, oleuropein also reduced the cytotoxicity in mutant ST*Hdh* ^*Q111/Q111*^ striatal cells.

### 3.2 Oleuropein reduces mutant htt-induced ROS accumulation

Increased ROS levels often lead to excess oxidization of cellular macromolecules, such as DNA, lipids, and proteins (Reichmann et al., 2018; Lévy et al., 2019; Yuan et al., 2021; Yu et al., 2024). Knowing that expression of mHtt protein is associated with increased ROS (Trettel et al., 2000; Milakovic et al., 2006) and oleuropein is a potent antioxidant (Kruk et al., 2005; Choi et al., 2021), we asked whether oleuropein affects the cellular redox state that subsequently reduces the cytotoxicity of the mHtt model cells. To test this, the cellular ROS and superoxide (SO) levels were monitored in the ST*Hdh* ^*Q7/Q7*^ cells expressing the FLAG-tagged truncated mHtt protein with a 103 polyQ stretch (FLAG-QP103). Ectopic expression of the FLAG-QP103 protein caused an increase in ROS levels as compared to the control ST*Hdh* ^*Q7/Q7*^ striatal cells ectopically expressing the FLAG tagged-truncated wild-type Htt protein (FLAG-QP25) (from 29% to 45%; *p* = 0.019, black bars in Figure 1D) and SO (from 25% to 42%; *p* = 0.0091, red bars in Figure 1D). Furthermore, oleuropein significantly decreased the ROS levels (from 45% to 35%; *p* = 0.029, black bars in Figure 1D) and SO levels (from 42% to 32%; *p* = 0.033, red bars in Figure 1D) in the ST*Hdh* ^*Q7/Q7*^ striatal cells ectopically expressing the mutant FLAG-QP103 protein. These results suggest that oleuropein may reduce the cytotoxicity of the HD model cells by decreasing the cellular ROS and SO levels.

We then asked whether oleuropein acts on modulating ROS levels to reduce the cytotoxicity of the mutant ST*Hdh* ^*Q111/Q111*^ striatal cells. As predicted, the mutant ST*Hdh* ^*Q111/Q111*^ striatal cells had increased ROS signals as compared to those of the wild-type ST*Hdh^Q7/Q7^* striatal cells (from 25% to 37%; *p* = 0.0062, black bars in Figure 1E). Consistently, oleuropein treatment significantly reduced the percentage of cells with positive ROS signals in the mutant ST*Hdh* ^*Q111/Q111*^ striatal cells (from 37% to 27%; *p* = 0.017, black bars in Figure 1E). Oleuropein treatment also significantly reduced the percentage of mutant ST*Hdh* ^*Q111/Q111*^ striatal cells with SO signals (from 34% to 25%; *p* = 0.048, red bars in Figure 1E). Taken together, our data demonstrated that cytotoxicity alleviation correlates with the reduction of cellular ROS and SO levels in all mHtt models tested.

### 3.3 Oleuropein pre-treatment protects against acute oxidative stress in HD model cells

Because of the ability of oleuropein to reduce ROS and SO accumulation in mHtt model cells, we asked whether oleuropein could protect neurons from oxidative stress. To this end, acute oxidative stress was induced in the ST*Hdh* cells treated with 100 μM tBHP for 4 h. After tBHP treatment, the cell viability of ST*Hdh^Q7/Q7^* striatal cells decreased compared to the untreated cells (from 100% to 77.65%; *p* = 0.0001; Figure 1F), indicating cells were under oxidative stress. We then tested the effect of oleuropein on the ST*Hdh^Q7/Q7^* striatal cells treated with tBHP. The ST*Hdh^Q7/Q7^* striatal cells were preincubated with oleuropein at different concentrations for 24 h before challenging with tBHP. We found that pre-incubation with 5 μg/mL oleuropein increased the viability of tBHP-challenged ST*Hdh^Q7/Q7^* striatal cells (from 77.65% to 92.06%; *p* = 0.0013; Figure 1F), suggesting that oleuropein protects neuronal cells from acute oxidative stress.

It is reported that HD striatal neurons are sensitive to oxidative stress (Jalgaonkar et al., 2023). In addition, the accumulation of mHtt aggregates is associated with increased ROS levels, reduced mitochondrial function, and decreased cell viability (Yin et al., 2016; Soares et al., 2019; Fão and Rego, 2021). Knowing that oleuropein protects the ST*Hdh* ^*Q7/Q7*^ striatal cells from tBHP-induced acute oxidative stress, we were interested in testing whether oleuropein could protect mutant ST*Hdh* ^*Q111/Q111*^ striatal cells, which already had relatively high ROS levels (Figure 1E), from additional acute oxidative stress. To this end, the mutant ST*Hdh* ^*Q111/Q111*^ striatal cells were pre-incubated with oleuropein at the concentration of 0.5 μg/mL, 2 μg/mL, or 5 μg/mL for 24 h before subjecting to 100 μM tBHP treatment. As predicted, the tBHP-treated mutant ST*Hdh* ^*Q111/Q111*^ striatal cells had lower cell viability as compared to the tBHP-treated ST*Hdh^Q7/Q7^* striatal cells (from 100% to 83.41%; *p* = 0.005; Figure 1G). Pre-incubation with 5 μg/mL oleuropein, on the other hand, protected mutant ST*Hdh* ^*Q111/Q111*^ striatal cells from tBHP-induced oxidative stress (from 83.41 to 96.96; *p* = 0.0183; Figure 1G). Oleuropein at the concentration of 0.5 μg/mL or 2 μg/mL did not have effect. Taken together, our data support that oleuropein at the concentration of 5 μg/mL is capable of protecting ST*Hdh^Q7/Q7^* striatal cells and the mutant ST*Hdh* ^*Q111/Q111*^ striatal cell from acute oxidative stress.

### 3.4 Oleuropein reduces mutant huntingtin aggregates

**FIGURE 2:**
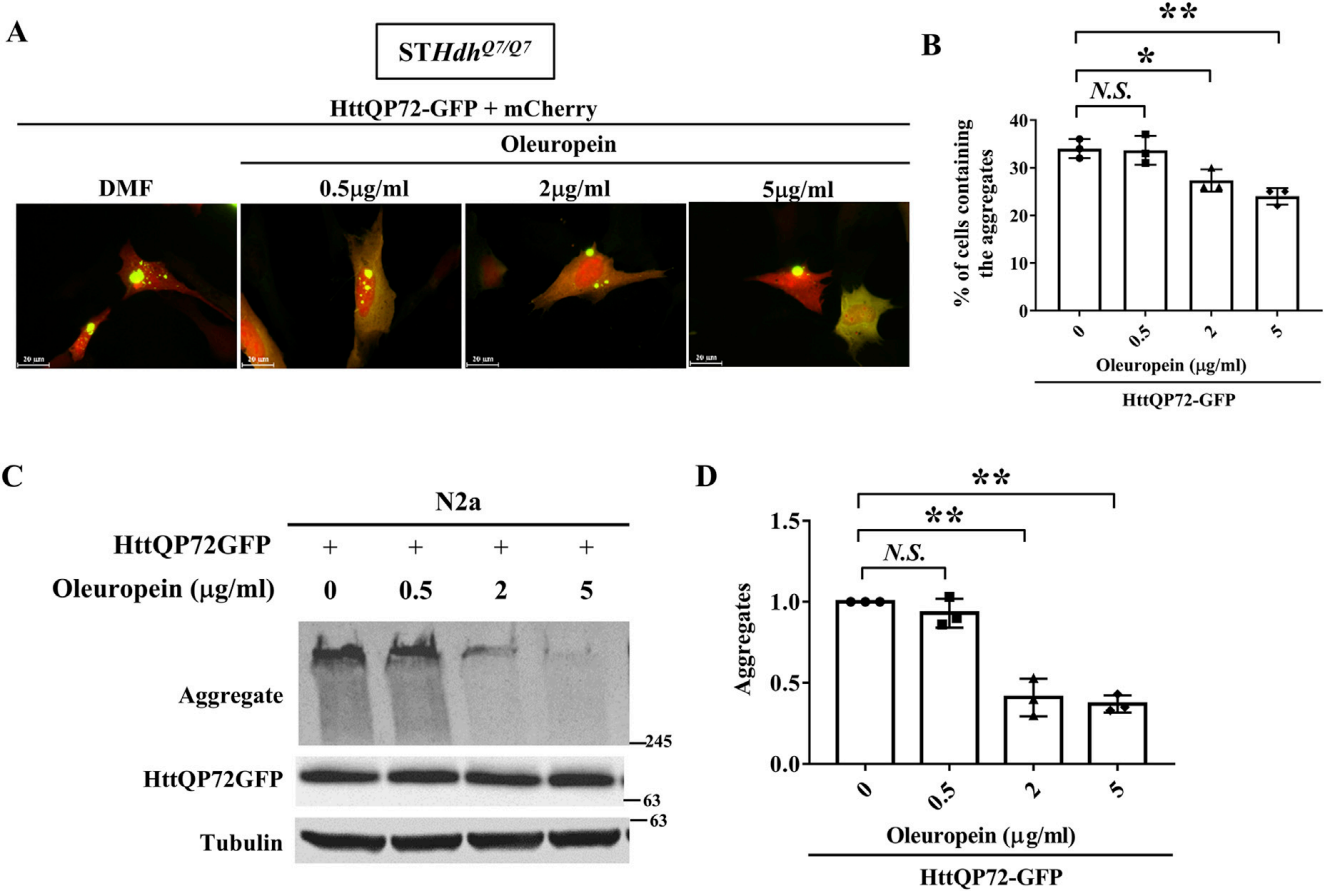
Oleuropein reduces mutant huntingtin aggregates. **(A)** Images showing ST*Hdh* ^*Q7/Q7*^ striatal cells co-transfected with the HttQP72-GFP and mCherry plasmids after oleuropein treatment. Cells without treatment were used as the control. **(B)** Quantification analysis of the percentage of cells containing the aggregates in ST*Hdh* ^*Q7/Q7*^ striatal cells transfected with the indicated plasmids in the absence or presence of oleuropein treatment at the indicated concentration for 24 h. **(C)** Immunoblot detection of the aggregated and soluble forms of HttQP72-GFP in N2a cells transfected with the indicated plasmid incubated in the absence or presence of oleuropein at the indicated concentration for 24 h. Tubulin protein was used as an internal control. **(D)** Quantification analyses of the normalized HttQP72-GFP aggregates. Tubulin was used as an internal control for normalization in **(C)**. Data from three independent experiments are presented as mean normalized units ± SEM. Data showing significant differences are labeled as follow: *p* < 0.05 with one asterisk (*), and *p* < 0.01 with two asterisks (**). *N.S.*, no significance.

Accumulation of mHtt aggregates is a major factor that leads to the death of striatal neurons and is recognized as one of the main pathogenic factors in HD (Davies et al., 1997; DiFiglia et al., 1997; Martín-Aparicio et al., 2001; Intihar et al., 2019; Layburn et al., 2022). Because oleuropein protects the ST*Hdh^Q7/Q7^* striatal cells ectopically expressing the truncated mHtt protein and also reduced mHtt-associated ROS accumulation (Figure 1), we asked whether oleuropein-mediated alleviation of HD pathology is linked to decrease in the mHtt aggregates. To test this idea, we monitored the HttQP72-GFP aggregates in the ST*Hdh* ^*Q7/Q7*^ striatal cells ectopically expressing the truncated mutant HttQP72-GFP protein in the absence or presence of oleuropein at different concentrations for 24 h. Oleuropein at the concentration of 2 μg/mL and 5 μg/mL caused a visible decrease in the accumulation of HttQP72-GFP aggregates (Figure 2A). Quantification analysis also confirmed that oleuropein treatment significantly reduced the percentage of cells containing the HttQP72-GFP aggregates (from 34% to 27% at the concentration of 2 μg/mL and from 34% to 24% at the concentration of 5 μg/mL); *p* = 0.047 and *p* = 0.0072, respectively, Figure 2B).

Oleuropein-mediated reduction of HttQP72-GFP aggregates was also confirmed in N2a cells by immunoblotting (Figure 2C). Quantification analysis also confirmed oleuropein significantly reduced the amount of HttQP72-GFP aggregates (from one to 0.4 at the concentration of 2 μg/mL and from one to 0.37 at the concentration of 5 μg/mL; *p* = 0.0065 and *p* = 0.0053, respectively, Figure 2D). Taken together, our data indicate that oleuropein reduces the accumulation of HttQP72-GFP aggregates.

### 3.5 Oleuropein reduces the soluble form of mHtt in mutant huntingtin-expressing cells

**FIGURE 3:**
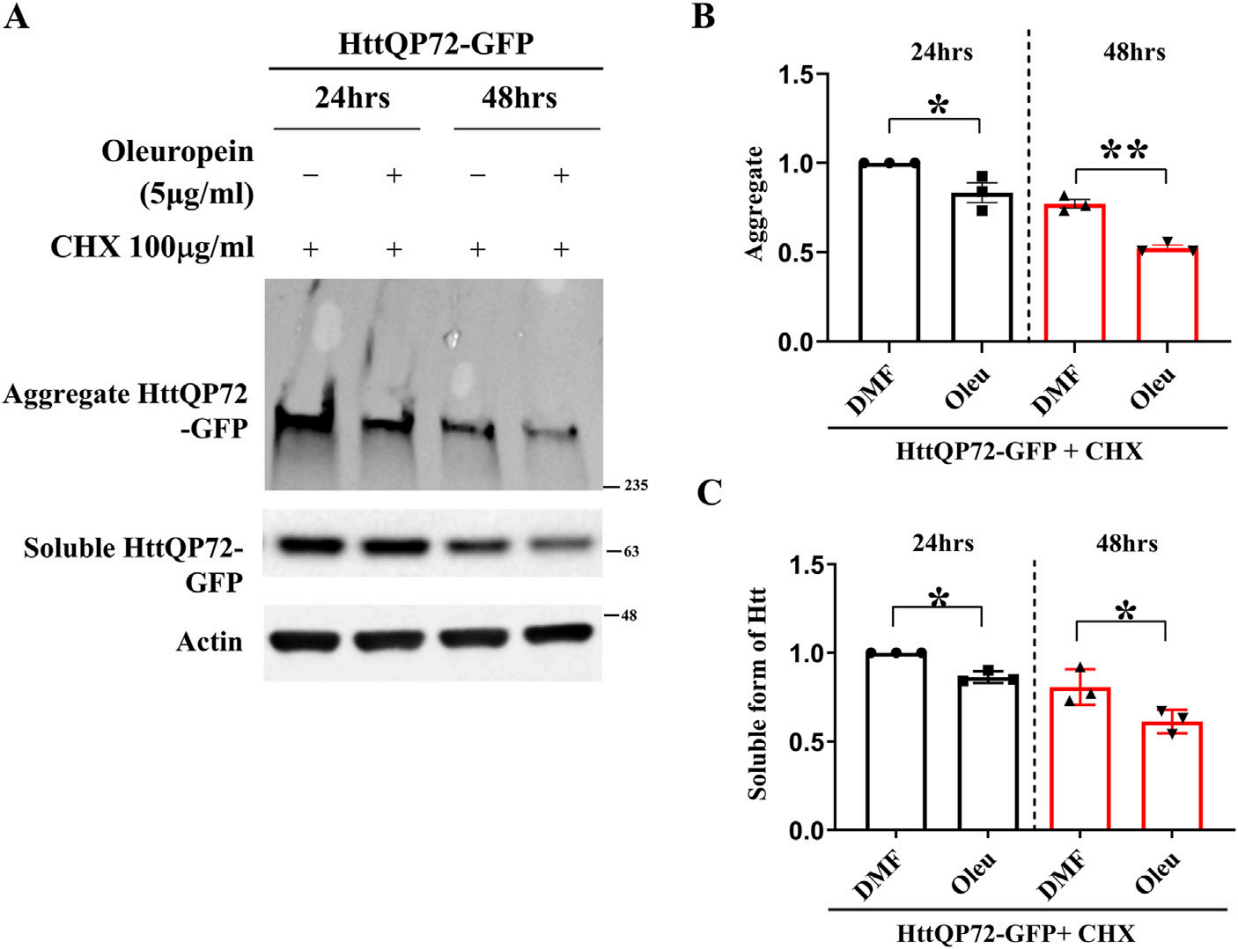
Oleuropein decreases the aggregated and soluble forms of truncated mHtt in N2a cells. **(A)** Immunoblot detection of the aggregated and soluble forms of mutant Htt (HttQP72-GFP) in N2a cells transfected with the HttQP72-GFP plasmid in the absence or presence of oleuropein treatment at the indicated concentration for 24 or 48 h. Cells were treated with 100 μg/ml cycloheximide (CHX) to inhibit protein synthesis. Actin was used as an internal control. **(B)** Quantification analyses of the aggregated form of HttQP72-GFP protein. **(C)** Quantification analyses of the soluble form of HttQP72-GFP protein. Actin was used for normalization. Data from three independent experiments are presented as mean normalized units ± SEM. Data showing significant differences are labeled as follows: *p* < 0.05 with one asterisk (*) and *p* < 0.01 with two asterisks (**).

Reducing the amount of soluble mHtt is pivotal to preventing the formation of huntingtin oligomers and facilitating the dissolution of mHtt aggregates in HD (Valionyte et al., 2020). Since oleuropein reduces the accumulation of mHtt aggregates, we asked whether oleuropein affects the stability and overall abundance of mHtt protein. To address this, soluble mutant HttQP72-GFP protein was monitored in the N2a cells treated with oleuropein. The N2a cells were treated with cycloheximide to prevent *de novo* protein synthesis. As expected, oleuropein treatment decreased mutant HttQP72-GFP protein aggregation at 24 h (from 1 to 0.83-fold, *p* = 0.0404) and 48 h (from 0.77 to 0.52-fold, *p* = 0.0011) post-treatment (Figures 3A, B). Additionally, the soluble form of mutant HttQP72-GFP protein was also reduced 24 h (from 1 to 0.86-fold; *p* = 0.0033) and 48 h (from 0.81 to 0.6-fold, *p* = 0.048) after oleuropein treatment (Figures 3A, C). The effect of oleuropein on the soluble form of mHtt protein was also tested in ST*Hdh* ^*Q7/Q7*^ striatal cells. Consistently, oleuropein reduced the soluble form of the mutant HttQP72-GFP protein 24 h (from 1 to 0.7033-fold; *p* = 0.0365) and 48 h (from 1.05 to 0.6767-fold; *p* = 0.0163) after treatment (Supplementary Figure S2A).

Since oleuropein reduces the soluble form of mutant HttQP72-GFP protein, we asked whether oleuropein has a similar effect on the stability and overall abundance of the full-length mHtt protein. To address this question, the endogenous full-length mHtt protein was monitored in oleuropein-treated ST*Hdh* ^*Q111/Q111*^ striatal cells. Cycloheximide was added to prevent *de novo* protein synthesis. We found that oleuropein treatment did not affect the level of endogenous full-length mHtt protein (Supplementary Figure S3A). Similarly, oleuropein treatment did not affect the transiently expressed full-length mHtt protein level in ST*Hdh* ^*Q111/Q111*^ striatal cells (Supplementary Figure S3C). Hence, our results indicated that the effect of oleuropein on the soluble form of truncated mHtt protein appeared to be specific.

### 3.6 Oleuropein does not affect autophagic flux in the HD model cells

**FIGURE 4:**
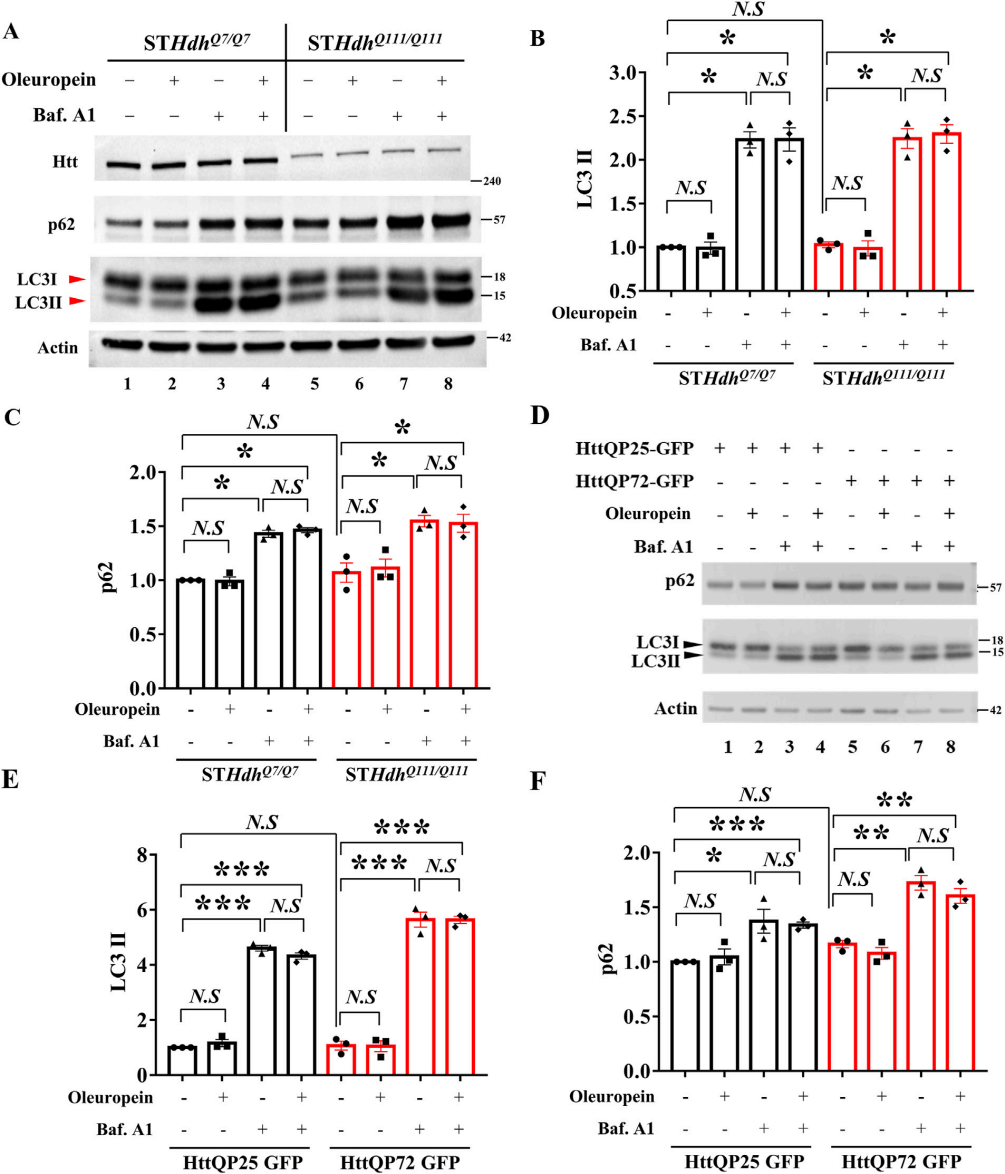
Oleuropein does not affect autophagic flux in HD model cells. **(A)** Immunoblotting analysis of the ST*Hdh* ^*Q7/Q7*^ and ST*Hdh* ^*Q111/Q111*^ striatal cells treated with 5 μg/ml oleuropein or 200 nM Bafilomycin A1 or both. Immunoblot detection of Htt, LC3, and p62 proteins in ST*Hdh* ^*Q7/Q7*^ and ST*Hdh* ^*Q111/Q111*^ striatal cells. Actin was used as an internal control. Quantitation analyses of **(B)** LC3-II or **(C)** p62 protein normalized to actin. **(D)** Immunoblotting analysis of LC3 and p62 in N2a cells expressing truncated wild-type or mutant Htt after treatment with 5 μg/ml oleuropein or 200 nM Bafilomycin A1 or both. Actin protein was used as an internal control. Quantitation analysis of **(E)** LC3II or **(F)** p62 protein normalized to actin. Data from three independent experiments are presented as mean normalized units ± SEM. Data showing significant differences are labeled as follows: *p* < 0.05 with one asterisk (*), *p* < 0.005 with two asterisks (**), and *p* < 0.001 with three asterisks (***). *N.S.*, not significant.

The Autophagy-Lysosome pathway and the Ubiquitin-Proteasome system are the endogenous mechanisms to reduce mutant Htt aggregates (Valionyte et al., 2020). Because oleuropein aglycone, a deglycosylated derivative of oleuropein, has been reported to induce autophagy (Rigacci et al., 2015; Achour et al., 2016; Miceli et al., 2018; Leri et al., 2021), we tested whether oleuropein activates the autophagy pathway to reduce mHtt aggregates. To do this, the autophagy markers, LC3-II, and p62, were monitored in wild-type ST*Hdh^Q7/Q7^* striatal cells and mutant ST*Hdh^Q111/Q111^* striatal cells by immunoblotting analysis. Our result showed that oleuropein treatment did not affect the LC3-II and p62 protein levels in wild-type ST*Hdh^Q7/Q7^* striatal cells and those in the mutant ST*Hdh* ^*Q111/Q111*^ striatal cells (Q7: LC3II from 1 to 0.99-fold; *p* > 0.05; p62 from 1 to 0.99-fold; *p* > 0.05, Figures 4A–C, lane 1 and 2. Q111: LC3II from 1.03 to 0.9867-fold; *p* > 0.05; p62 from 1.07 to 1.113-fold; *p* > 0.05; Figures 4A–C, land 5 and 6). Because the autophagic flux is attributed to both induction and degradation of autophagosome by lysosome that cannot be simply differentiated through examining the steady state of LC3-II and p62 levels [reviewed in Klionsky et al. (2021)]. Alternatively, we used Bafilomycin A1 to block lysosomal degradation and examined whether the accumulation of LC3-II and p62 proteins is affected by oleuropein (Huang et al., 2021; Klionsky et al., 2021). As the control, Bafilomycin A1 treatment increased accumulation of the LC3-II protein in wild-type ST*Hdh^Q7/Q7^* striatal cells (from 1 to 2.23-fold; *p* = 0.0002, Figures 4A, B, lanes 1 and 3) and the mutant ST*Hdh* ^*Q111/Q111*^ striatal cells (from 1.03 to 2.243 folds; *p* = 0.0005, Figures 4A, B, lanes 5 and 7), indicating lysosomal degradation was inhibited. After Bafilomycin A1 treatment, oleuropein did not increase the accumulation of LC3-II protein further in the wild-type ST*Hdh^Q7/Q7^* striatal cells (from 2.23 to 2.233 folds; *p* > 0.05, Figures 4A, B, lanes 3 and 4) or mutant ST*Hdh* ^*Q111/Q111*^ striatal cells (from 2.243 to 2.297 folds; *p* > 0.05, Figures 4A, B, lanes 7 and 8), indicating oleuropein did not increase the autophagic flux. Consistently, Bafilomycin A1 increased accumulation of the p62 protein in wild-type ST*Hdh^Q7/Q7^* striatal cells (from 1 to 1.43 folds; *p* = 0.0002, Figures 4A, C, lanes 1 and 3) and the mutant ST*Hdh* ^*Q111/Q111*^ striatal cells (from 1.07 to 1.547 folds; *p* = 0.0101, Figures 4A, C, lanes 5 and 7), confirming lysosomal degradation was inhibited. Oleuropein treatment failed to increase the accumulation of p62 protein in the Bafilomycin A1-treated wild-type ST*Hdh^Q7/Q7^* striatal cells (from 1.43 to 1.463 folds; *p* > 0.05, Figures 4A, C, lanes 3 and 4) and Bafilomycin A1-treated mutant ST*Hdh* ^*Q111/Q111*^ striatal cells (from 1.547 to 1.527 folds; *p* > 0.05, Figures 4A, C, lanes 7 and 8). Hence, our data indicate that oleuropein treatment does not induce autophagy in the ST*Hdh* HD striatal model cells.

To investigate whether the lack of oleuropein effect on autophagy is cell-specific or a general phenomenon, the effect of oleuropein on the autophagic flux was also examined in the neuronal N2a cells. Similar to the results observed in the ST*Hdh* striatal model cells, oleuropein did not affect the LC3-II and p62 protein levels in N2a cells ectopically expressing the truncated wild-type HttQP25-GFP or the mutant HttQP72-GFP protein (QP25:LC3II from 1 to 1.165-fold; *p* > 0.05; p62 from 1 to 1.045-fold; *p* > 0.05, Figures 4D–F, lane 1 and 2. QP72: LC3II from 1.07 to 1.048-fold; *p* > 0.05; p62 from 1.162 to 1.078-fold; *p* > 0.05; Figures 4D–F, land 5 and 6), indicating that overall autophagy balance was not affected by oleuropein. Bafilomycin A1-dependent increase of the LC3-II protein in the neuronal N2a cells ectopically expressing truncated wild-type HttQP25-GFP (from 1 to 4.607 folds; *p* < 0.0001, Figures 4D, E, lanes 1 and 3) or mutant HttQP72-GFP protein (from 1.07 to 5.642 folds; *p* = 0.0001, Figures 4D, E, lanes 5 and 7). After blocking lysosomal degradation by Bafilomycin A1, oleuropein did not affect the LC3-II protein level in N2a cells overexpressing either the truncated wild-type HttQP25-GFP protein (from 4.6 to 4.32 folds; *p* > 0.05, Figures 4D, E, lanes 3 and 4) or the truncated mutant HttQP72-GFP protein (from 5.64 to 5.63 folds; *p* > 0.05, Figures 4D, E, lanes 7 and 8). Similarly, blocking lysosomal degradation did not affect the p62 protein level in the N2a cells overexpressing either the truncated wild-type HttQP25-GFP protein (from 4.6 to 4.32 folds; *p* > 0.05, Figures 4D, F, lanes 3 and 4) or the truncated mutant HttQP72-GFP protein (from 5.64 to 5.63 folds; *p* > 0.05, Figures 4D, F, lanes 7 and 8). In summary, our data indicate oleuropein did not act through the autophagy pathway to relieve the cytotoxicity of the HD model cells.

### 3.7 Oleuropein increases the proteolytic activity of the 20S proteasome

**FIGURE 5:**
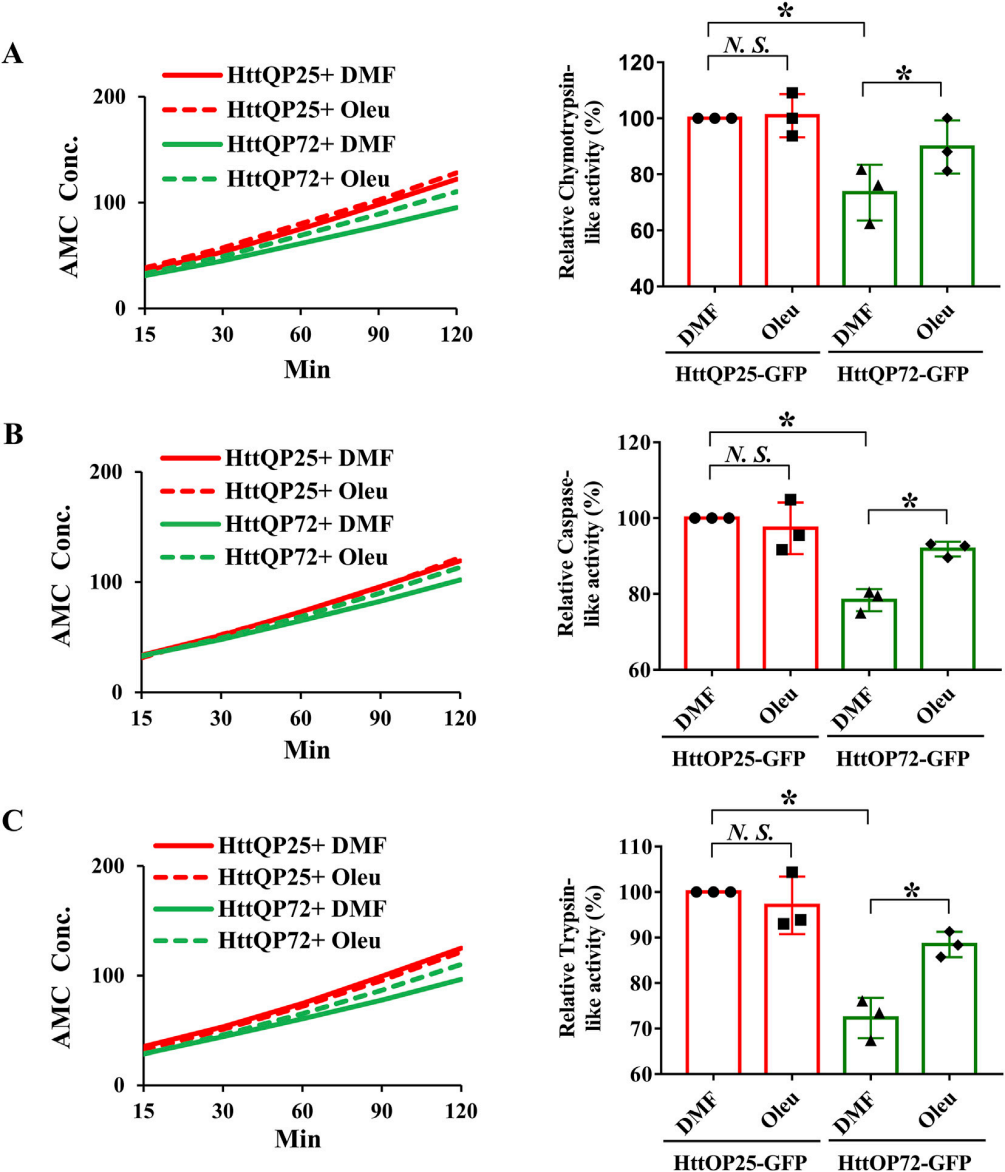
Oleuropein increases the proteasome activity in N2a cells expressing the truncated HttQP72-GFP mHtt protein. Oleuropein increases the proteasome activity in N2a cells expressing the truncated HttQP72-GFP mHtt protein. The chymotrypsin-like activity **(A)**, caspase-like activity **(B)**, and trypsin-like activity **(C)** were examined in N2a cells expressing wild-type or the truncated mHtt in the absence or presence of 5 μg/ml oleuropein treatment at the indicated concentration for 24 h. Data from three independent experiments are presented as mean normalized units ± SEM. Data showing a significant difference (*p* < 0.05) is labeled with one asterisk (*). *N.S.*, not significant.

Because oleuropein did not affect the autophagy pathway, we tested whether the proteasome system was activated in oleuropein-treated HD model cells. Because the substrate-specific activities of the chymotrypsin-like, trypsin-like and caspase-like proteases are the major proteolytic activity of the 20S core proteasome complex [reviewed in Wang and Le (2019); Wang et al. (2020)], we examined the chymotrypsin-like, caspase-like and trypsin-like activities in neuronal N2a cells ectopically expressing the truncated wild-type HttQP25-GFP protein or truncated mutant HttQP72-GFP protein in the absence or presence of oleuropein treatment. Consistent with the previous report (Huang et al., 2021), ectopic expression of the mutant HttQP72-GFP protein decreased the activities of the chymotrypsin-like protease (from 100% to 73%; *p* = 0.043, Figure 5A), caspase-like protease (from 100% to 78%; *p* = 0.037, Figure 5B) and trypsin-like protease (from 100% to 72%; *p* = 0.029, Figure 5C). Consistent with the previous reports (Katsiki et al., 2007; Trader et al., 2017), oleuropein treatment increased the activities of chymotrypsin-like protease (from 73% to 90%; *p* = 0.017, Figure 5A), caspase-like protease (from 78% to 92%; *p* = 0.026, Figure 5B), and trypsin-like protease (from 72% to 88%; *p* = 0.024, Figure 5C) in cells expressing the mutant HttQP72-GFP protein. In contrast, oleuropein did not alter the activities of chymotrypsin-like protease (100%–101%; *p* > 0.05, Figure 5A), caspase-like protease (from 100% to 97%; *p* > 0.05, Figure 5B), and trypsin-like protease (from 100% to 97%; *p* > 0.05, Figure 5C) in cells expressing the HttQP25-GFP protein.

In addition to the N2a cells, we also tested the effect of oleuropein on proteasome activity in the ST*Hdh* striatal cells. As compared to the wild-type ST*Hdh^Q7/Q7^* striatal cells, the mutant ST*Hdh* ^*Q111/Q111*^ striatal cells had reduced activities of the chymotrypsin-like protease (from 100% to 68%; *p* = 0.028, Supplementary Figure S4A), caspase-like protease (from 100% to 74%; *p* = 0.012, Supplementary Figure S4B), and trypsin-like protease (from 100% to 76%; *p* = 0.014, Supplementary Figure S4C). Importantly, oleuropein treatment was able to significantly increase the activities of chymotrypsin-like protease (from 68% to 87%; *p* = 0.011, Supplementary Figure S4A), caspase-like protease (from 74% to 88%; *p* = 0.027, Supplementary Figure S4B), and trypsin-like protease (from 76% to 89%; *p* = 0.034, Supplementary Figure S4C) in the mutant ST*Hdh* ^*Q111/Q111*^ striatal cells. Similar to the N2a cells, oleuropein did not affect the chymotrypsin-like activity (100%–103%; *p* > 0.05, Supplementary Figure S4A), caspase-like activity (from 100% to 102%; *p* > 0.05, Supplementary Figure S4B), and trypsin-like activity (from 100% to 102%; *p* > 0.05, Supplementary Figure S4C) in the wild-type ST*Hdh^Q7/Q7^* striatal cells. Taken together, our data showed that oleuropein could modulate 20S proteasome proteolytic activity in the N2a and ST*Hdh* HD model cells.

### 3.8 Oleuropein affects the proteasome pathway via an unidentified mechanism

**FIGURE 6:**
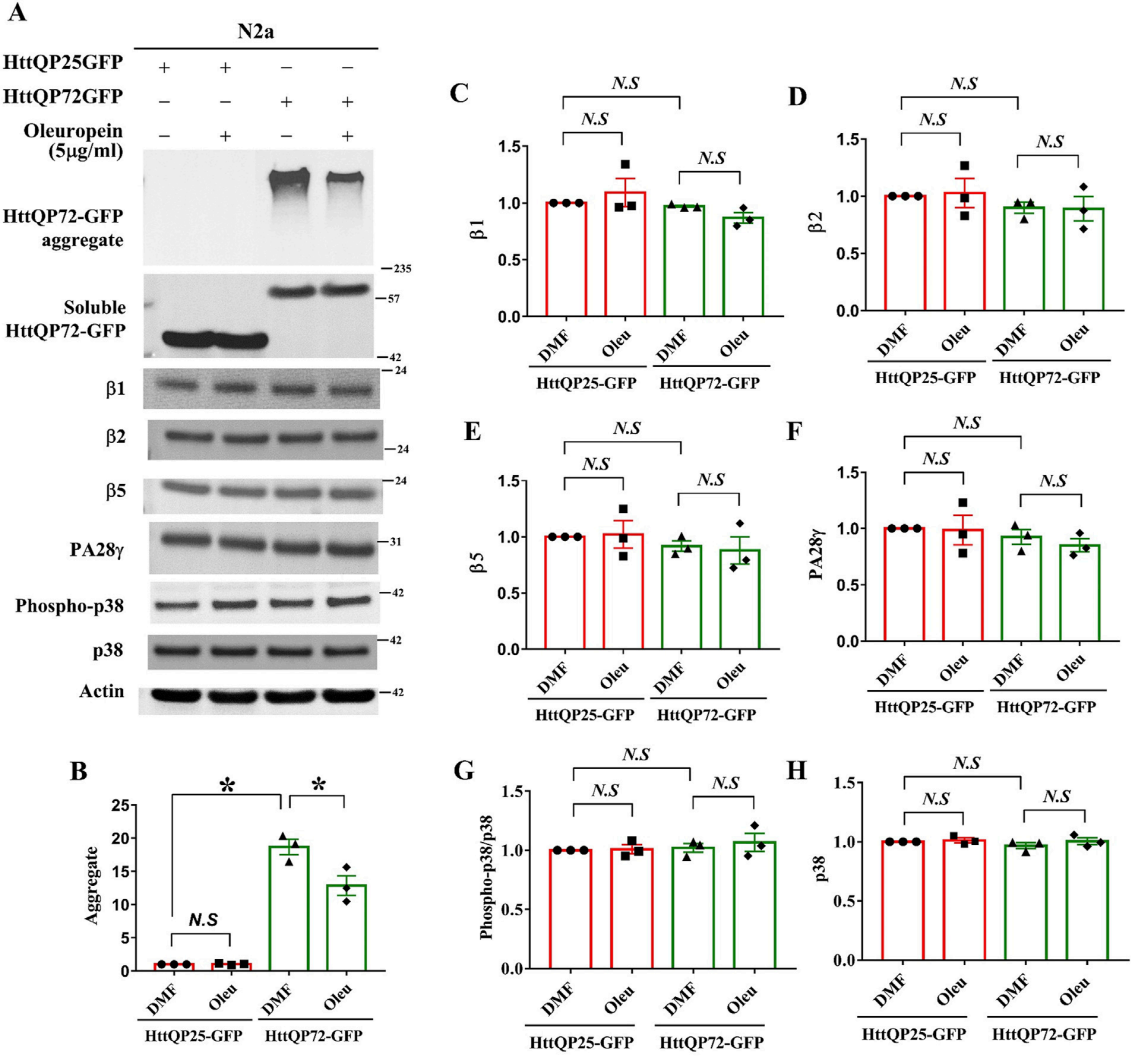
Effects of oleuropein on the proteasome-associated proteins in N2a cells expressing the truncated HttQP72-GFP mHtt protein. **(A)** Immunoblot detection of Htt, β1, β2, β5, PA28γ, phospho-p38, and p38 in N2a cells expressing truncated wild-type or mutant Htt in the absence or presence of oleuropein treatment at the indicated concentration for 24 h. Actin was used as an internal control. **(B–H)** Quantification analyses of protein levels with the indicated protein normalized to the actin protein. A paired Student’s t-test was used for statistical analysis. Data showing significant differences compared to the control are labeled as follows: *p* < 0.05 with one asterisk (*), and *p* < 0.01 with two asterisks (**). *N.S.*, no significance.

To investigate how oleuropein affects the proteasome pathway, we used immunoblotting to examine the catalytic subunits of the proteasome pathway. Protein levels of subunits β1, β2, and β5 that represent the respective caspase-like, trypsin-like, and chymotrypsin-like proteases of 20S proteasome were examined in the N2a cells overexpressing the HttQP25-GFP protein or mutant HttQP72-GFP protein in the presence or absence of oleuropein. As the control, oleuropein treatment reduced the accumulation of HttQP72-GFP aggregates (from 18.68 to 12.87 folds; *p* = 0.0363, Figures 6A, B). Even though an ectopic expression of mutant HttQP72-GFP protein decreased activities of all three core proteases of the 20S proteasome (Figure 5), it did not affect the steady-state levels of β1 (Figures 6A, C), β2 (Figures 6A, D) or β5 protein (Figures 6A, E). Moreover, oleuropein treatment did not affect the levels of β1 (Figures 6A, C), β2 (Figures 6A, D), or β5 proteins (Figures 6A, E).

Ectopic expression of the proteasome activator PA28γ has been shown to improve cellular functions of the ST*Hdh* ^*Q7/Q7*^ striatal cells and motor behaviors in HD model mice (Seo et al., 2007; Jeon et al., 2016). Since the proteasomal subunits β1, β2, and β5 were not the targets for oleuropein-dependent proteasome activities, we asked whether the 20S proteasome activator, PA28γ (Cascio, 2021), was the target of oleuropein. Our results showed that neither expression of mutant HttQP72-GFP protein alone nor combination of oleuropein treatment and expression of mutant HttQP72-GFP protein affected PA28γ protein level in the N2a cells (Figures 6A, F), indicating PA28γ is not the target of oleuropein. As the control, the expression of HttQP25-GFP protein and its combination with oleuropein treatment did not affect the PA28γ protein (Figures 6A, F).

Activation of the p38 MAPK pathway has been shown to inhibit proteasome activity (Lee et al., 2010; Leestemaker et al., 2017; Huang et al., 2021). Consequently, we investigated whether oleuropein improved proteasome activity by inhibiting the p38 MAPK pathway. Ectopic expression of mutant HttQP72-GFP protein did not affect the level of phospho-p38 MAPK in the N2a cells (Figures 6A, G). Additionally, oleuropein treatment did not alter the ratio of phospho-p38 MAPK/p38 MAPK in cells expressing the HttQP72-GFP (Figures 6A, G, H), indicating that oleuropein did not affect the p38 MAPK pathway to enhance proteasome activities. The effect of oleuropein on the proteasome and p38 MAPK pathway was further tested in the ST*Hdh^Q7/Q7^* striatal cells. The catalytic subunits and regulator of the proteasome pathway were examined in ST*Hdh* ^*Q7/Q7*^ striatal cells expressing the mutant HttQP72-GFP protein. Consistent with the results from the N2a cells, oleuropein did not affect the protein levels of β1, β2, β5, or PA28γ in the ST*Hdh^Q7/Q7^* striatal cells expressing mutant HttQP72-GFP protein (Supplementary Figure S5A). Overexpression of mutant HttQP72-GFP protein increased the level of phospho-p38 MAPK in the ST*Hdh* ^*Q7/Q7*^ striatal cells, however, oleuropein did not affect the levels of unphosphorylated p38 MAPK or alter the p38 MAPK pathway, as indicated by the ratio of phospho-p38 MAPK to p38 MAPK (Supplementary Figure S5A).

We suspected that oleuropein might target other MAPK pathways. It has been shown that the Erk1/2 MAPK pathway promotes cell survival, while the c-Jun N-terminal kinase (JNK) MAPK pathway inhibits proteasome activity (Mitsiades et al., 2002; Tang et al., 2006; Shah and Orlowski, 2009). However, our experiments showed that oleuropein treatment did not affect either the Erk1/2 or the JNK pathway in the N2a cells expressing HttQP72-GFP (Supplementary Figure S6), suggesting that oleuropein does not act on the MAPK pathways to reduce mHtt aggregates. Consistently, oleuropein did not affect the protein levels of proteasome catalytic subunits β1, β2, and β5, proteasome activator PA28γ, or alter the MAPK pathways in both wild-type ST*Hdh^Q7/Q7^* and the mutant ST*Hdh* ^*Q111/Q111*^ striatal cells (Supplementary Figure S7 and Supplementary Figure S8).

Given that the rho-associated kinases (ROCKs) pathway has been reported to reduce soluble and aggregated forms of mHtt (Bauer and Nobuyuki, 2009; Bauer et al., 2009), we examined the effect of oleuropein on the ROCKs pathway. Based on our data, oleuropein treatment did not affect the ROCK pathway in N2a cells expressing HttQP25-GFP, as well as in both wild-type ST*Hdh^Q7/Q7^* and mutant ST*Hdh* ^*Q111/Q111*^ striatal cells (Supplementary Figure S9).

Nuclear factor erythroid 2-related factor 2 (Nrf2) is known to coordinate oxidative stress and proteasome functions by enhancing proteasomal activity, degrading oxidized protein, and maintaining proteostasis (Arlt et al., 2009). Since protein aggregation and dysfunction caused by oxidative stress often require 20S proteasome activity (Reichmann et al., 2018; Lévy et al., 2019), oleuropein may modulate the Nrf2 transcription factor to regulate cellular redox balance and proteasome activity. Interestingly, the Nrf2 pathway was activated in the mutant ST*Hdh* ^*Q111/Q111*^ striatal cells compared to the control ST*Hdh* ^*Q7/Q7*^ striatal cells, as evidenced by an increase in the ratio of phospho-Nrf2 to Nrf2 from 1 to 1.348-fold (*p* = 0.0014; Supplementary Figure S10). However, oleuropein treatment did not further activate the Nrf2 pathway, as indicated by no additional change in the phospho-Nrf2/Nrf2 ratio in both ST*Hdh* ^*Q111/Q111*^ and control ST*Hdh* ^*Q7/Q7*^ striatal cells (Supplementary Figure S10).

In summary, while oleuropein increased the proteolytic activities of the 20S proteasome in all three HD cell models, it did not do so by modulating the major proteasome catalytic subunit and its PA28γ activator, or the MAPK, ROCKS, and the Nrf2 pathways.

## 4 Discussion

The expression and accumulation of mHtt are crucial for inducing cell cytotoxicity and cell death in neuronal cell cultures (Saudou et al., 1998; Bjørkøy et al., 2005; Raspe et al., 2009). Strategies to reduce the accumulation of mHtt aggregates have been shown to improve cell viability and animal behavior in HD cell and animal models (Hodgson et al., 1999; Bates, 2003; Arrasate and Finkbeiner, 2012; Weiss et al., 2012). In this study, we provided compelling evidence to show that oleuropein reduced cytotoxicity in three HD cell models. Moreover, oleuropein is potent in lowering the soluble form of mHtt protein, mHtt aggregates, and associated ROS. Because the proteolytic activities of the proteasome pathway increased in oleuropein-treated cells, we proposed that oleuropein activates the proteasome pathway to reduce the truncated mHTT protein. However, oleuropein did not affect the core subunits and activator of the 20S proteasome, or the MAPK, ROCKs, and Nrf2 pathways. In addition to the MAPK, ROCKs, and Nrf2 pathways, the cAMP-dependent protein kinase (PKA) pathway has also been shown to inhibit the proteasome activity, specifically targeting the caspase- and trypsin-like proteasome activities (Zhang et al., 2007; Goldberg et al., 2021). Given that oleuropein increased all three protease activities, it is unlikely PKA is the target of oleuropein. This suggests that other regulatory mechanisms of the proteasome pathway are involved.

The question then became how oleuropein enhances proteasome activity. Oxidative stresses have been known to affect the 26S proteasome activity (Ding and Keller, 2001). It is possible that mHtt triggers oxidative stress that inhibits proteasome activities. The primary substrates of 20S proteasome degradation are misfolded proteins, and their accumulation can result in cytotoxicity (Ferrington et al., 2001; David et al., 2002; Shringarpure and Davies, 2002; Liu et al., 2003; Whittier et al., 2004; Baugh et al., 2009; Raynes et al., 2016). Increasing oxidative stress can lead to the overaccumulation of oxidatively damaged misfolded proteins, which overload the proteasome and inhibit its core protease activity (Pajares et al., 2015; Korovila et al., 2017). Oleuropein may enhance the proteasomal core protease activities through an unidentified mechanism, thereby reducing stress caused by mutant Htt protein. It will be essential to identify the mechanism via an unbiased approach.

Posttranslational modifications (PTMs) have been reported to modulate proteasome activity (Kors et al., 2019; Bi et al., 2021). Oxidative stress has been shown to increase O-GlcNAcylation and S-glutathionylation modification of the proteasome (Zachara and Hart, 2004; Zachara et al., 2004; Dalle-Donne et al., 2009; Xiong et al., 2011; Groves et al., 2013; Reeves et al., 2014). O-linked β-N-acetylglucosamine (O-GlcNAc) modification of the proteasomal 19S regulatory cap Rpt2 protein and S-glutathionylation of the proteasomal 19S regulatory cap RPN1 and RPN2 proteins are reported to reduce proteasome activity and lead to the accumulation of ubiquitylated protein (Zhang et al., 2003; Zmijewski et al., 2009; Keembiyehetty et al., 2011; Liu et al., 2014). Interestingly, upregulated O-GlcNAcylation and S-glutathionylation modified proteins have been reported to impair cell homeostasis and contribute to neuron loss in HD model mice and model cells (Yuzwa et al., 2008; Kumar et al., 2014; Hong et al., 2015; Grima et al., 2017; Lee et al., 2021). Therefore, oleuropein may modulate O-GlcNAcylation or S-glutathionylation modification of the proteasomal proteins to improve proteasome activity and relieve oxidative stress of the mHtt-expressing cells. Further studies are required to address this possibility.

In this study, we did not observe oleuropein’s effect on autophagy in three HD cell models. Oleuropein aglycone, the deglycosylated oleuropein, has been shown to activate autophagy in human neuroblastoma SH-SY5Y cells, rat pancreatic epithelial RIN5F cells, and the cortex of TgCRND8 AD mice (Rigacci et al., 2015; Leri et al., 2021). Although both oleuropein and oleuropein aglycone possess anti-oxidative, anti-inflammatory, and lipid-lowering properties, their distinct structures may contribute to different modes of action (Xu et al., 2018). Further experiments are required to elucidate the effects of oleuropein and oleuropein aglycone on autophagy and/or proteasome.

Even though oleuropein has been shown to have neuroprotective functions (Achour et al., 2016; Omar et al., 2018; Brunetti et al., 2020; Leri et al., 2021; Marianetti et al., 2022), identifying the specific targets through which oleuropein reduce cytotoxicity remains challenging. In this study, we showed that oleuropein-mediated protection is linked to reducing ROS levels. Oleuropein may act as a direct ROS scavenger to alleviate oxidative stress in the HD model cells. Since mitochondria are the primary source of ROS production, we speculate that mitochondria-associated ROS balance may be affected by oleuropein. Equally possible is that oleuropein may trigger an endogenous antioxidative response to reduce oxidative stress and protect the HD model cells.

The strategies to reduce soluble and aggregated forms of mHtt have been demonstrated to be essential to alleviate HD pathology (Zhu et al., 2019; Hegde et al., 2020; Valionyte et al., 2020; Srinivasan et al., 2022). Here, we showed that oleuropein potently decreases the soluble and aggregated forms of truncated mHtt protein and cytotoxicity. Our results suggest that oleuropein can be used as a potential therapeutic remedy. Moreover, we showed that oleuropein may target the proteasome pathway to improve neuronal health in HD model cells. We propose that the proteasome pathway may serve as the therapeutic target for drug discovery for Huntington’s disease.

## Data Availability

The original contributions presented in the study are included in the article/Supplementary Material, further inquiries can be directed to the corresponding author.

## Abbreviations

AMC, 7-amino-4-methylcoumarin; Baf, Bafilomycin A1; β1, proteasome 20S core caspase-like protease subunit; β2, proteasome 20S core trypsin-like protease subunit; β5, proteasome 20S core chymotrypsin-like protease subunit; CCK8, cell counting kit eight assay; CHX, cycloheximide; DMF, Dimethylformamide; Erk1/2 MAPK, extracellular signal-regulated mitogen-activated protein kinases 1/2; HD, Huntington’s disease; Htt, huntingtin; JNK MAPK, c-Jun N-terminal mitogen-activated protein kinases; LC3, Microtubule-associated protein 1A/1B-light chain 3; mHtt, mutant huntingtin; Nrf2, Nuclear factor-erythroid 2 (NF-E2)-related factor 2; Oleu, oleuropein; p38 MAPK, p38 mitogen-activated protein kinases; p62, Sequestosome-1; PA28γ, proteasome activator subunit 3 (PSME3, Ki); PBS, Phosphate-buffered saline; Q7, ST*Hdh* ^*Q7/Q7*^ striatal cells; Q111, ST*Hdh* ^*Q111/Q111*^ striatal cells; ROCK, rho-associated kinases; ROS, reactive oxidative species; SO, superoxide; SEM, standard error of the mean; tBHP, tert-Butyl Hydroperoxide.
