# Supplementary material for: Oleuropein enhances proteasomal activity and reduces mutant huntingtin-induced cytotoxicity
Source: Front Pharmacol. 2024 Sep 13;15:1459909. doi: 10.3389/fphar.2024.1459909 (PMC11440197; doi:10.3389/fphar.2024.1459909)
Supplement: Supplementary file 1 [file DataSheet1.PDF]

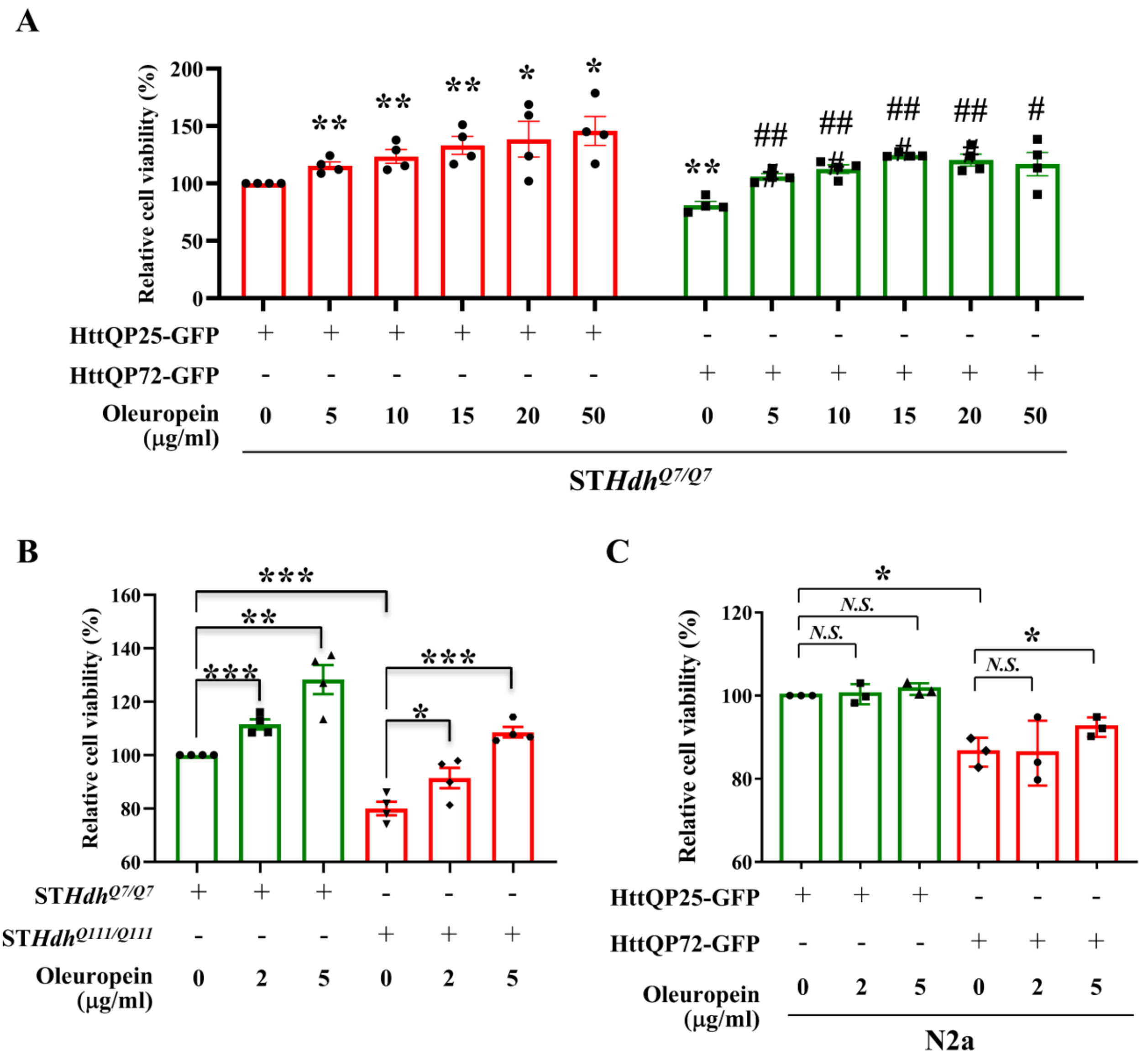

Figure S1. Oleuropein ameliorates mHtt-induced cytotoxicity.

(A) Cell viability analysis of ST*Hdh*$^{Q7/Q7}$ striatal cells transfected with the HttQP25-GFP or HttQP72-GFP plasmid. Forty-eight hours post-transfection, the cells were treated with oleuropein at the specified concentration for an additional 48 hours. Data showing a significant difference compared to the HttQP25-GFP transfected ST*Hdh*$^{Q7/Q7}$ striatal cells treated with 0 μg/ml oleuropein treatment is labeled with an asterisk (*); Data showing a significant difference compared to the HttQP72-GFP transfected ST*Hdh*$^{Q7/Q7}$ striatal cells treated with 0 μg/ml oleuropein treatment is labeled with a pound sign (#). (B) Cell viability analysis of ST*Hdh*$^{Q7/Q7}$ striatal cells and ST*Hdh*$^{Q111/Q111}$ striatal cells after a 48-hour incubation with oleuropein at the indicated concentration. (C) Cell viability analysis of N2a cells transfected with the HttQP25-GFP or HttQP72-GFP plasmid. Twenty-four hours post-transfection, the cells were treated with oleuropein at the indicated concentration for an additional 24 hours. Cells without treatment were used as the negative control. Data from three independent experiments are presented as mean normalized units ± SEM. Data showing significant differences are labeled as follows: $p < 0.05$ with one asterisk (*****), $p < 0.01$ with two asterisks (******), $p < 0.005$ with three asterisks (*******).

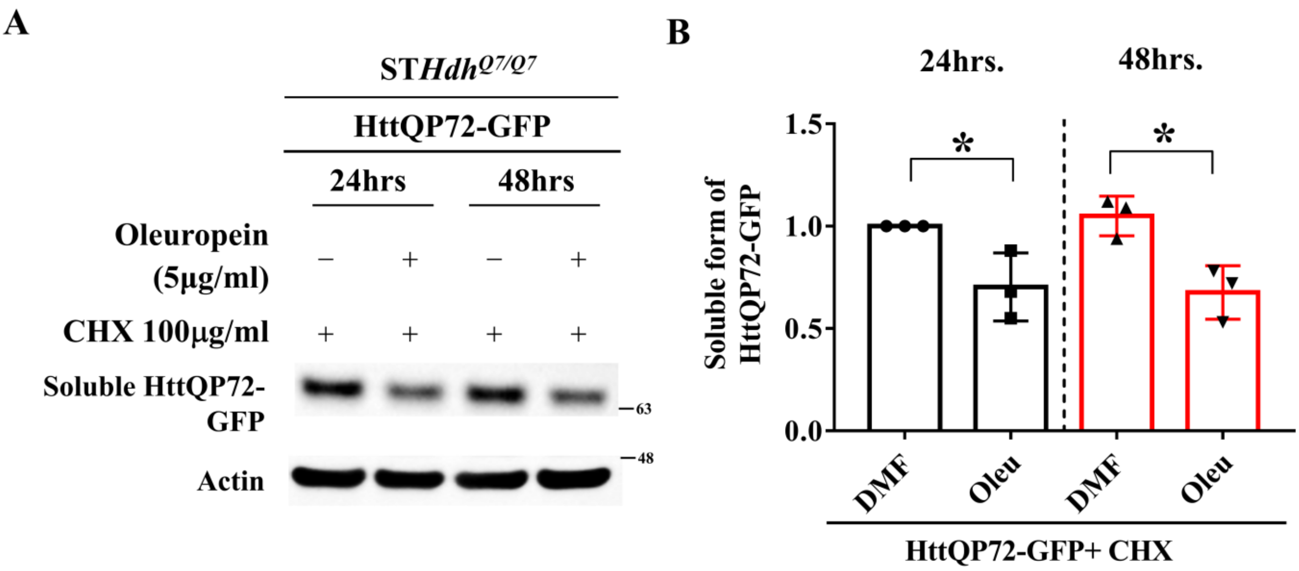

Figure S2. Oleuropein decreases the soluble form of truncated mHtt in ST*Hdh*$^{Q7/Q7}$ striatal cells.

(A) Immunoblot detection of the soluble form of mutant Htt (HttQP72-GFP) in ST*Hdh*$^{Q7/Q7}$ striatal cells transfected with the HttQP72-GFP plasmid in the absence or presence of oleuropein treatment at the indicated concentration for 24 or 48 hours. To block protein synthesis, cells were treated with 100 µg/ml cycloheximide (CHX). Actin protein was used as an internal control. (B) Quantification analyses of the soluble form of HttQP72-GFP protein. Actin was used for normalization. Data are from three independent experiments and presented as mean normalized units ± SEM. Data showing a significant difference ($p < 0.05$) is labeled with one asterisk (*).

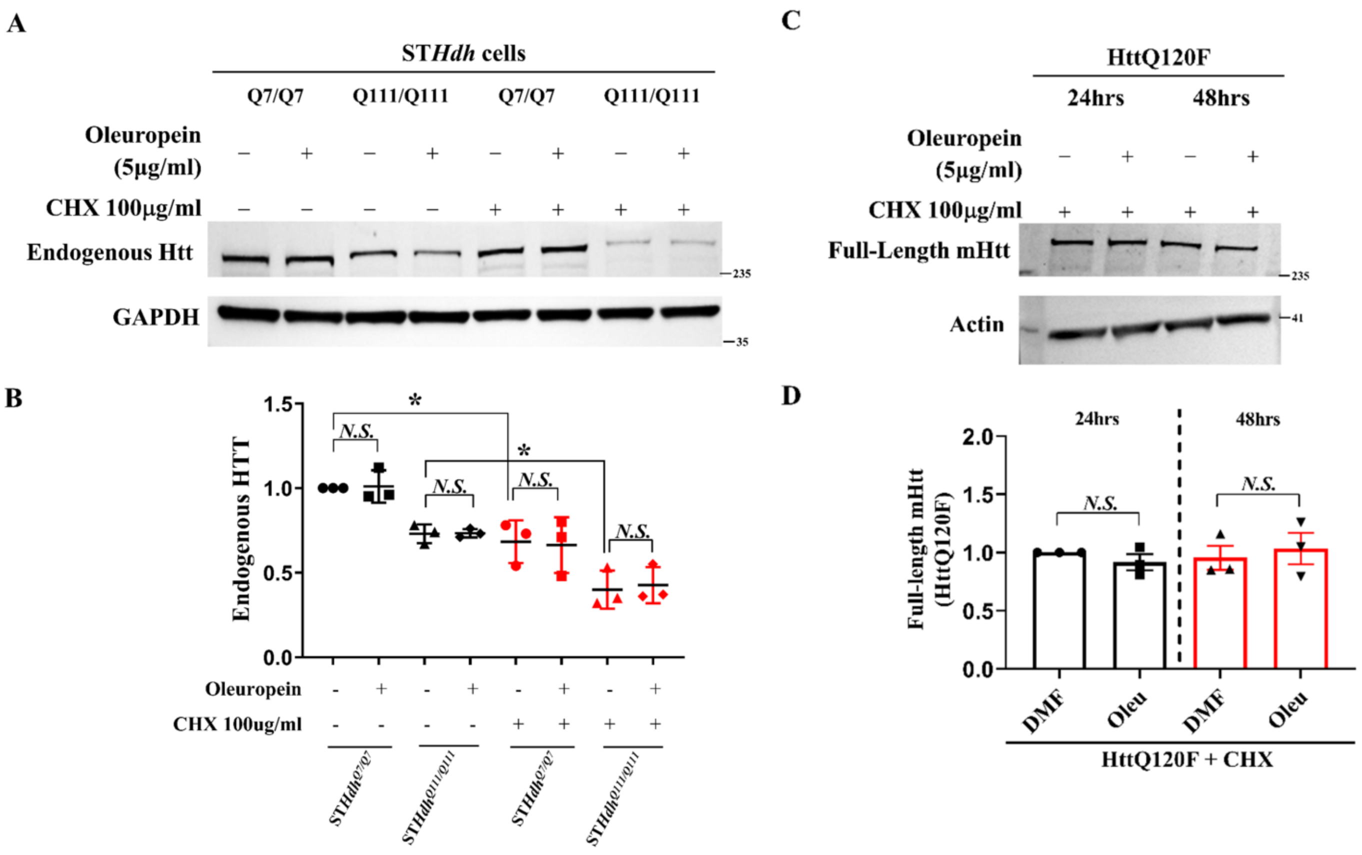

Figure. S3 Oleuropein did not affect the level of full-length mHtt in mutant ST*Hdh*$^{Q111/Q111}$ striatal cells.

(A) Immunoblot detection of endogenous full-length Htt and GAPDH proteins in ST*Hdh*$^{Q7/Q7}$ cells and ST*Hdh*$^{Q111/Q111}$ in the absence or presence of 5 µg/ml oleuropein treatment for 24 hours. Cells were treated with or without 100 µg/ml cycloheximide (CHX) to inhibit protein synthesis. (B) Quantification analyses of the endogenous Htt. GAPDH was used as an internal control for normalization in (B). (C) Immunoblot detection of the full-length mutant Htt (HttQ120F) and actin in ST*Hdh*$^{Q111/Q111}$ striatal cells transfected with the pEBV-HttQ120F plasmid in the absence or presence of 5 µg/ml oleuropein treatment for 24 or 48 hours. Cells were treated with 100 µg/ml cycloheximide (CHX) to inhibit protein synthesis. (D) Quantification analyses of the transfected full-length mutant Htt (HttQ120F). Actin was used for normalization. Data are from three independent experiments and presented as mean normalized units ± SEM. Data showed significant differences with $p < 0.05$ are labeled with one asterisk (*); N.S., no significance.

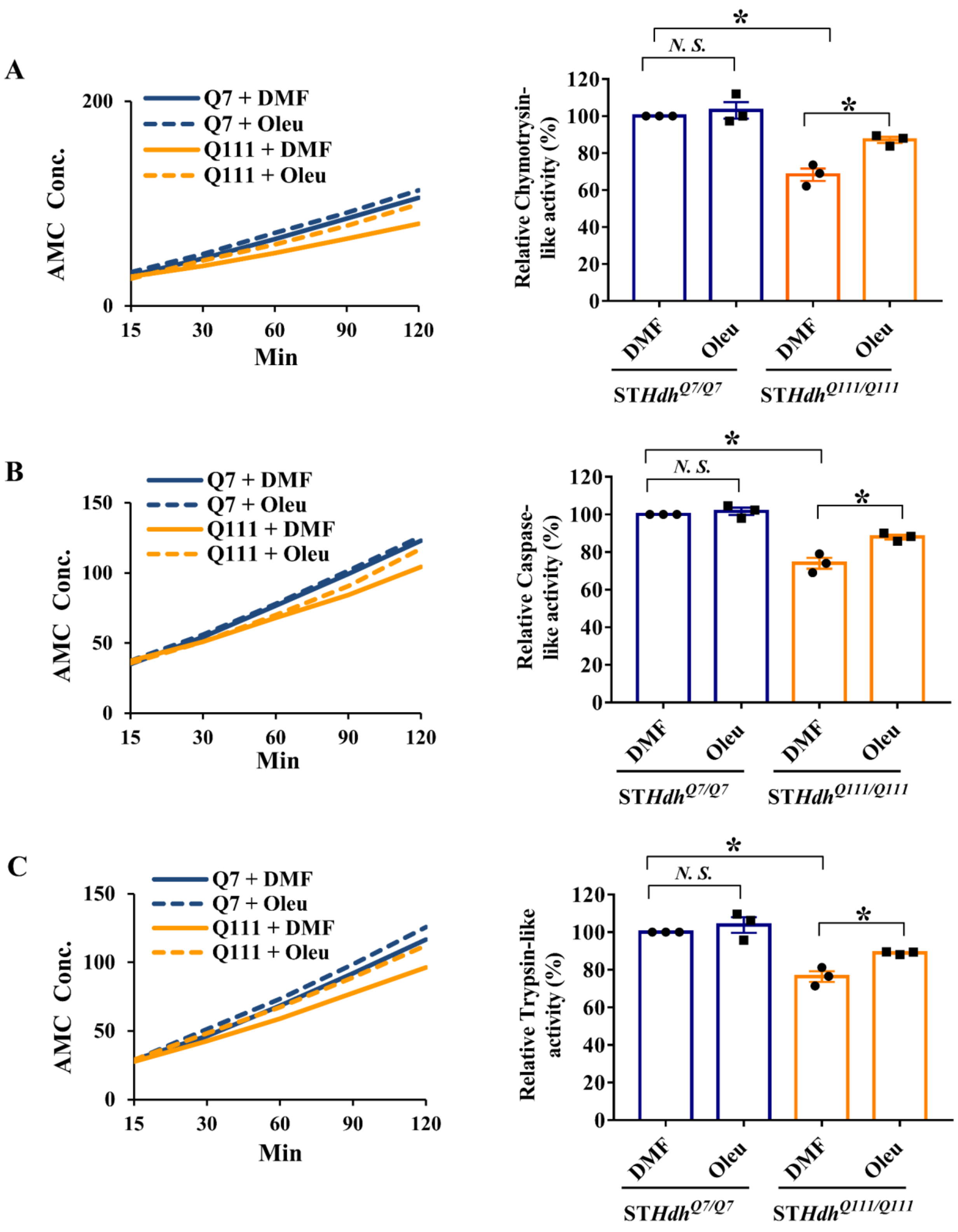

Figure S4. Oleuropein increases proteasome activity in mutant ST*Hdh*$^{Q111/Q111}$ striatal cells.

The chymotrypsin-like activity (A), caspase-like activity (B), and trypsin-like activity (C) were examined in ST*Hdh*$^{Q7/Q7}$ and ST*Hdh*$^{Q111/Q111}$ striatal cells in the absence or presence of oleuropein treatment at 5 µg/ml for 24 hours. Data are from three independent experiments and presented as mean normalized units ± SEM. Data showing significant differences with $p < 0.05$ are labeled with one asterisk (*); *N.S.*, not significant.

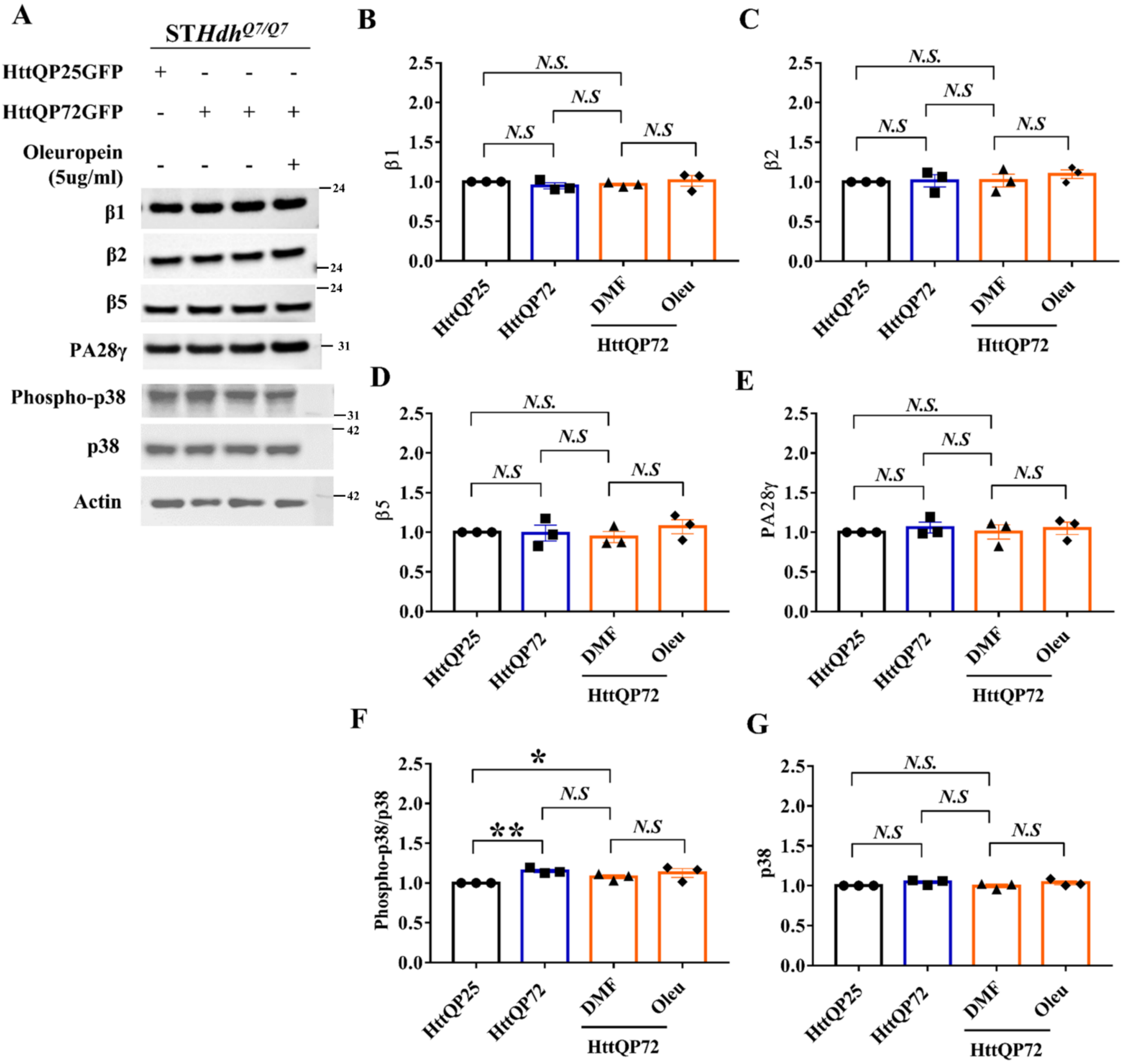

Figure S5. Oleuropein treatment does not affect p38 MAPK pathway in ST*Hdh*$^{Q7/Q7}$ striatal cells.

(A) Immunoblot detection of β1, β2, β5, PA28γ, phospho-p38, p38, and actin in ST*Hdh*$^{Q7/Q7}$ striatal cells expressing wild-type or the truncated mHtt in the absence or presence of oleuropein treatment at the indicated concentration for 24 hours. Actin was used as an internal control. (B)–(G) Quantification analyses on protein levels with the indicated protein normalized to the actin protein. A paired Student's t-test was used for statistical analysis. Data showing significant differences compared to control are labeled as follows: $P < 0.05$ with one asterisk (*), and $p < 0.01$ with two asterisks (**). *N.S.*, no significance.

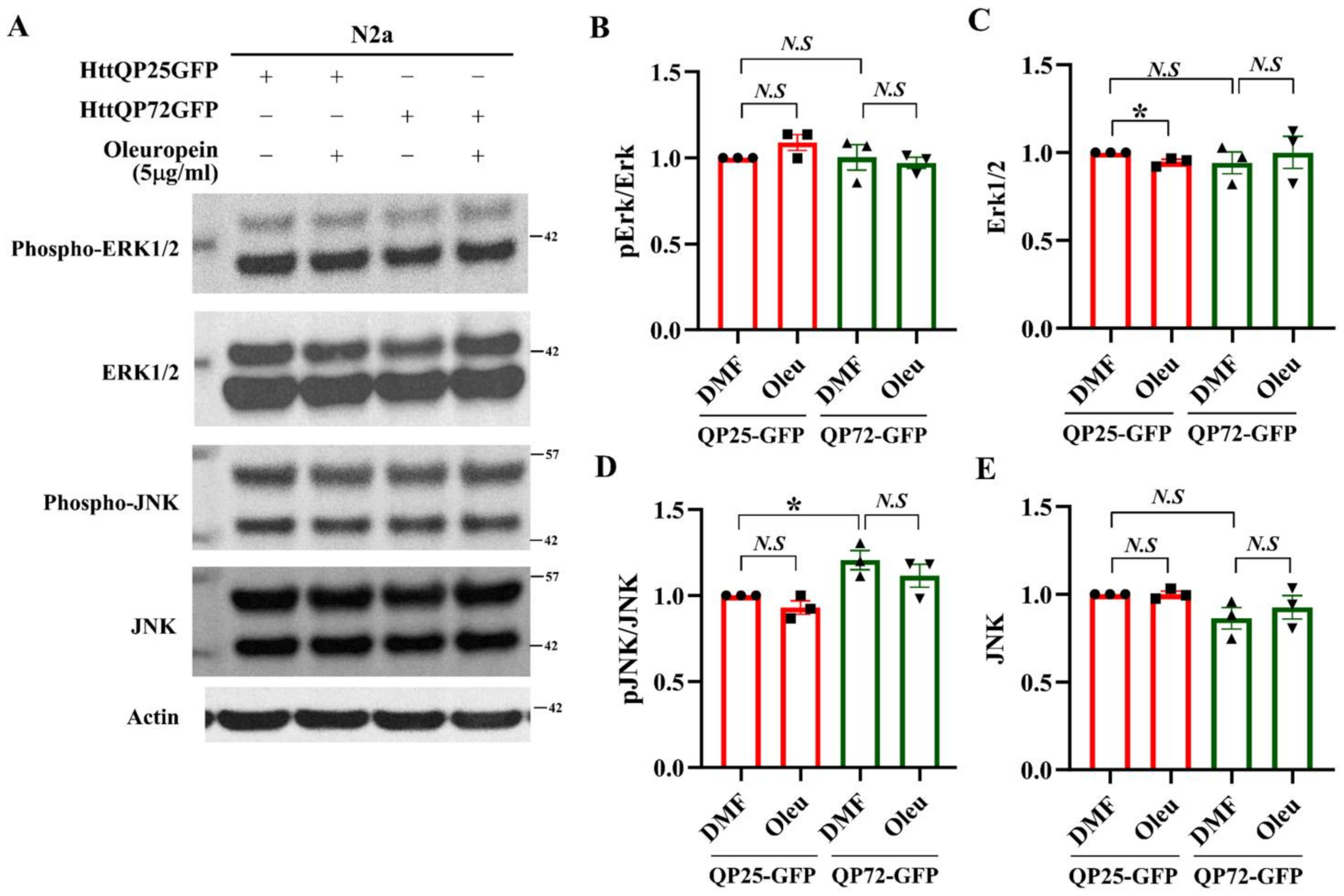

Figure S6. Oleuropein treatment does not affect the MAPK pathway in both wild-type and mutant Htt-expressing N2a cells.

(A) Immunoblot detection of phospho-Erk1/2, Erk1/2, phospho-JNK, JNK, and actin in N2a cells expressing either wild-type or truncated mHtt, with or without oleuropein treatment at the indicated concentration for 24 hours. (B-E) Quantification analyses on protein levels with the indicated protein normalized to the actin protein. A paired Student's t-test was used for statistical analysis. Data from three independent experiments are presented as mean normalized units ± SEM. Data showing a significant difference ($p < 0.05$) is labeled with one asterisk (*); *N.S.*, no significance.

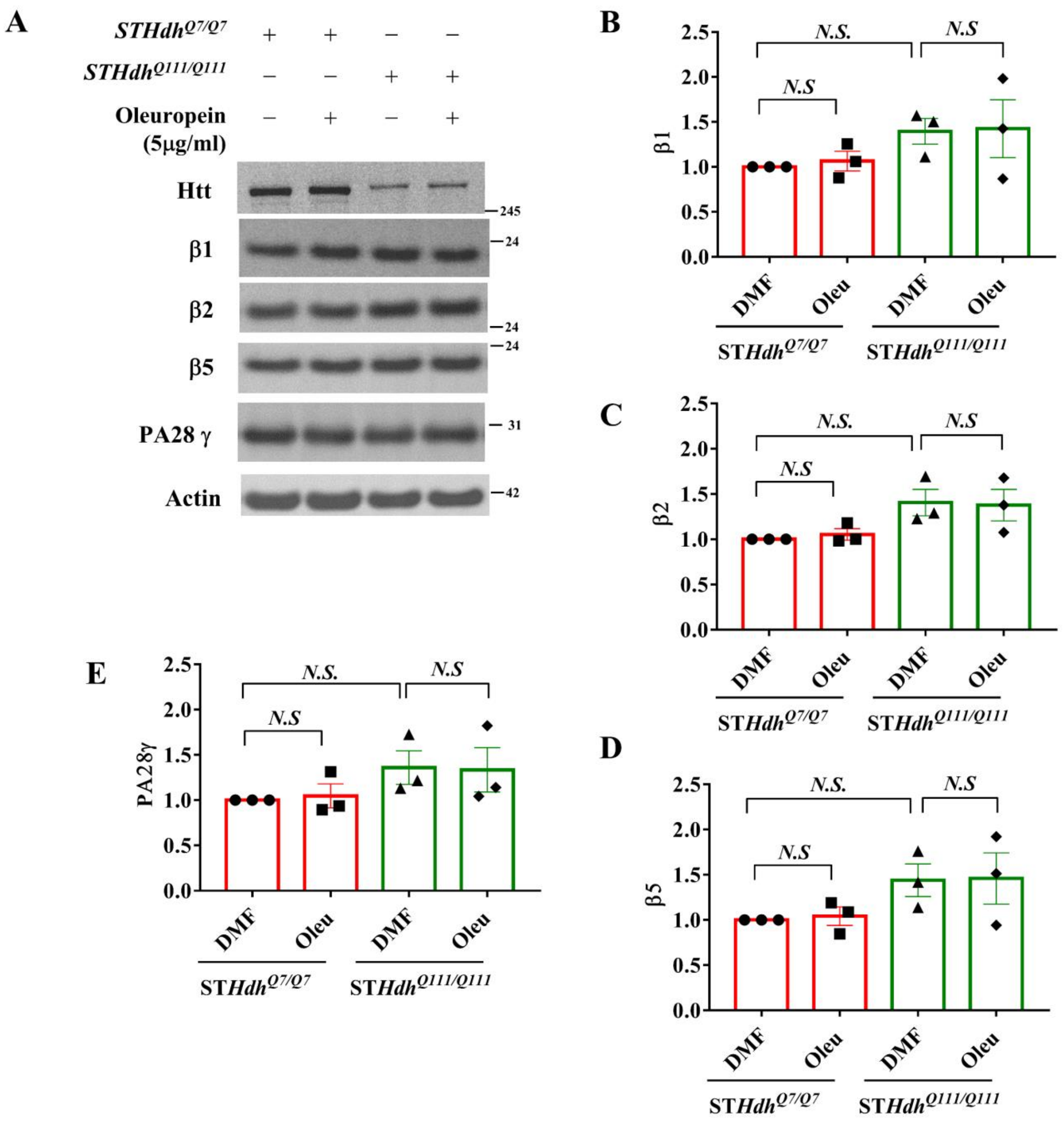

Figure S7. Effects of oleuropein on UPS-associated proteins in wild-type ST*Hdh*$^{Q7/Q7}$ and mutant ST*Hdh*$^{Q111/Q111}$ striatal cells.

(A) Immunoblot detection of Htt, β1, β2, β5, PA28γ, and actin in ST*Hdh*$^{Q7/Q7}$ and ST*Hdh*$^{Q111/Q111}$ striatal cells in the absence or presence of oleuropein treatment at the indicated concentration for 24 hours. (B)–(E) Quantification analyses on protein levels with the indicated protein normalized to the actin protein. A paired Student's t-test was used for statistical analysis. Data showing significant differences compared to control are labeled as follows: $p < 0.05$ with one asterisk (*), and $p < 0.01$ with two asterisks (**). *N.S.*: no significance.

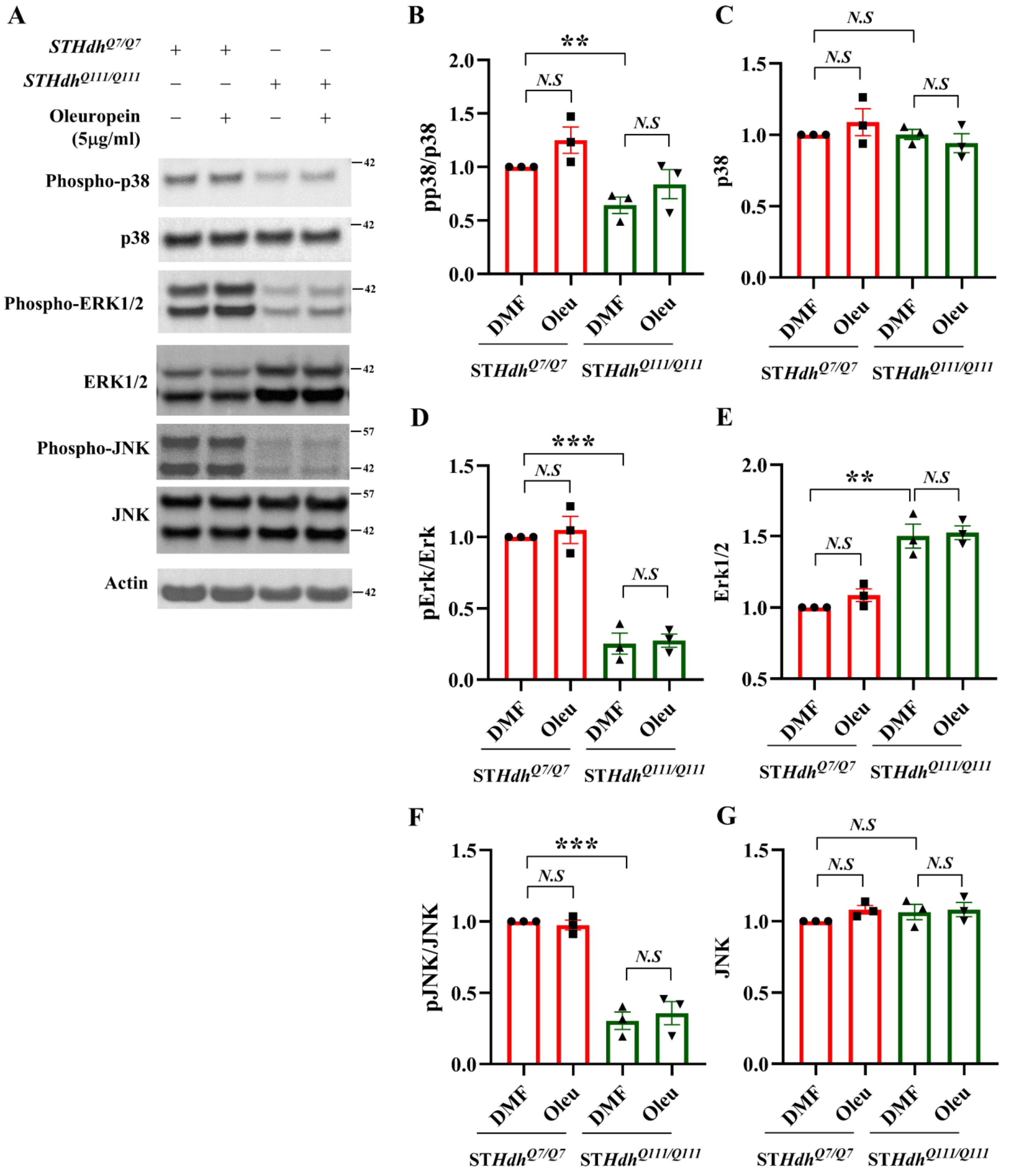

Figure S8. Oleuropein treatment does not affect the MAPK pathway in wild-type ST*Hdh*$^{Q7/Q7}$ and mutant ST*Hdh*$^{Q111/Q111}$ striatal cells.

(A) Immunoblot detection of phospho-p38, p38, phospho-Erk1/2, Erk1/2, phospho-JNK, JNK, and actin in wild-type ST*Hdh*$^{Q7/Q7}$ and mutant ST*Hdh*$^{Q111/Q111}$ striatal cells, with or without oleuropein treatment at indicated concentration for 24 hours. (B-G) Quantification analyses on protein levels with the indicated protein normalized to the actin protein. A paired Student's t-test was used for statistical analysis. Mutant ST*Hdh*$^{Q111/Q111}$ striatal cells had lower levels of phospho-p38 MAPK, phospho-Erk1/2 MAPK, and phospho-JNK MAPK (pp38/38: from 1 to 0.6426-fold; $p = 0.0099$; pErk1/2/Erk1/2: from 1 to 0.2525-fold; $p = 0.0005$; pJNK/JNK: from 1 to 0.3034-fold; $p = 0.0003$). Data are from three independent experiments and presented as mean normalized units ± SEM. Data showing significant differences are labeled as follows: $p < 0.01$ with two asterisks (**), and $p < 0.001$ with three asterisks (***). *N.S.*, no significance.

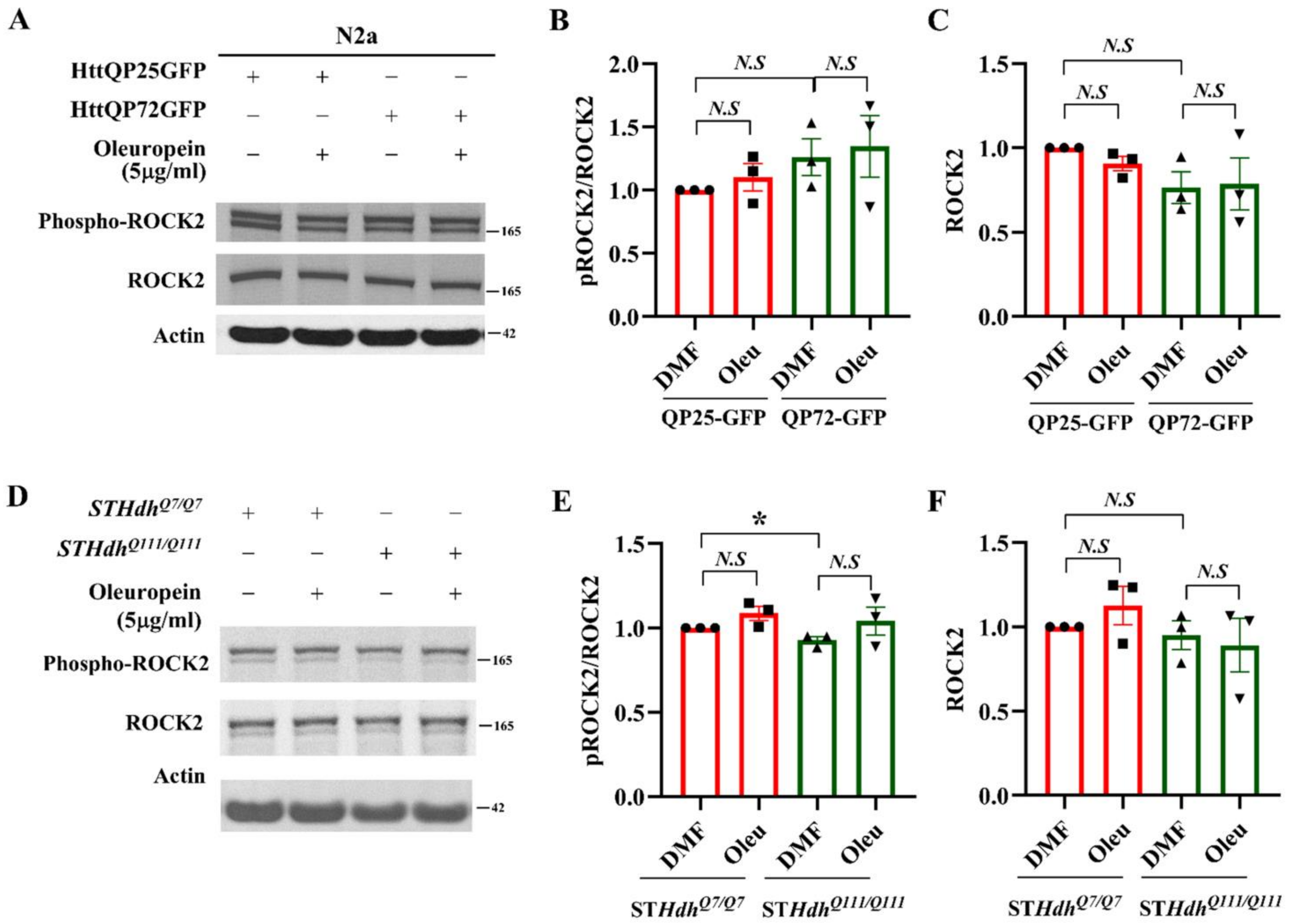

Figure S9. Oleuropein treatment does not affect the ROCK2 pathway in mHtt-expressing cells.

Immunoblot detection of phospho-ROCK2, ROCK2, and actin in (A) N2a cells expressing wild-type or the truncated mHtt (D) ST*Hdh*$^{Q7/Q7}$ and ST*Hdh*$^{Q111/Q111}$ striatal cells, with or without oleuropein treatment at the indicated concentration for 24 hours. (B)(C)(E)(F) Quantification analyses on protein levels with the indicated protein normalized to the actin protein. A paired Student's t-test was used for statistical analysis. Data from three independent experiments are presented as mean normalized units ± SEM. Data showing significant differences ($p < 0.05$) is labeled with one asterisk (*). *N.S.*, no significance.

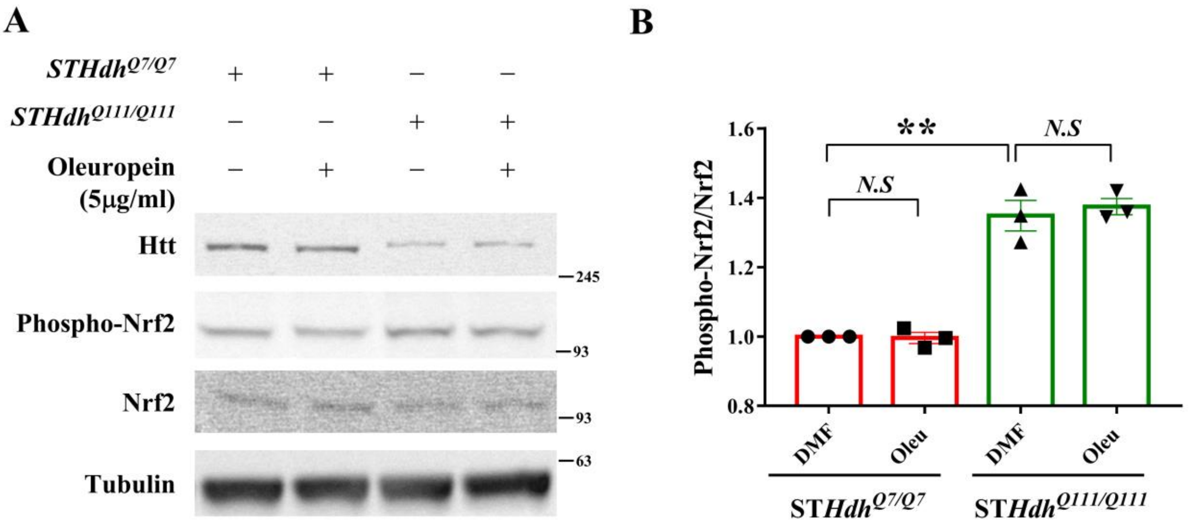

Figure S10. Oleuropein treatment does not affect Nrf2 pathway in ST*Hdh* striatal cells.

Immunoblot detection of Htt, phospho-Nrf2, Nrf2, and tubulin in ST*Hdh*$^{Q7/Q7}$ and ST*Hdh*$^{Q111/Q111}$ striatal cells in the absence or presence of oleuropein treatment at the indicated concentration for 24 hours. (B) Quantification analyses on protein levels with the indicated protein normalized to the tubulin protein. A paired Student's t-test was used for statistical analysis. Data from three independent experiments are presented as mean normalized units ± SEM. Data showing a significant difference ($p < 0.01$) is labeled with two asterisks (**); *N.S.*, no significance.
